# Exploring novel dilazep derivatives as hENT1 inhibitors and potentially covalent molecular tools

**DOI:** 10.1007/s11302-024-10026-x

**Published:** 2024-06-15

**Authors:** Majlen A. Dilweg, Marina Gorostiola González, Martijn D. de Ruiter, Nadine J. Meijboom, Jacobus P. D. van Veldhoven, Rongfang Liu, Willem Jespers, Gerard J. P. van Westen, Laura H. Heitman, Adriaan P. IJzerman, Daan van der Es

**Affiliations:** 1 Division of Drug Discovery and Safety, Leiden Academic Centre for Drug Research, Leiden University, PO Box 9502, Leiden, The Netherlands; 2 Oncode Institute, Leiden, The Netherlands

**Keywords:** Solute carrier, Human equilibrative nucleoside transporter 1, Dilazep, Structure–activity relationship, Covalent inhibitor

## Abstract

The human equilibrative nucleoside transporter 1 (SLC29A1, hENT1) is a solute carrier that modulates the passive transport of nucleosides and nucleobases, such as adenosine. This nucleoside regulates various physiological processes, such as vasodilation and -constriction, neurotransmission and immune defense. Marketed drugs such as dilazep and dipyridamole have proven useful in cardiovascular afflictions, but the application of hENT1 inhibitors can be beneficial in a number of other diseases. In this study, 39 derivatives of dilazep’s close analogue ST7092 were designed, synthesized and subsequently assessed using [^3^H]NBTI displacement assays and molecular docking. Different substitution patterns of the trimethoxy benzoates of ST7092 reduced interactions within the binding pocket, resulting in diminished hENT1 affinity. Conversely, [^3^H]NBTI displacement by potentially covalent compounds **14b**, **14c**, and **14d** resulted in high affinities (K_i_ values between 1.1 and 17.5 nM) for the transporter, primarily by the ability of accommodating the inhibitors in various ways in the binding pocket. However, any indication of covalent binding with amino acid residue C439 remained absent, conceivably as a result of decreased nucleophilic residue reactivity. In conclusion, this research introduces novel dilazep derivatives that are active as hENT1 inhibitors, along with the first high affinity dilazep derivatives equipped with an electrophilic warhead. These findings will aid the rational and structure-based development of novel hENT1 inhibitors and pharmacological tools to study hENT1's function, binding mechanisms, and its relevance in (patho)physiological conditions.

**Supplementary Information:**

The online version contains supplementary material available at 10.1007/s11302-024-10026-x.

## Introduction

The in- and efflux of nucleosides, nucleobases and nucleoside-derived xenobiotics in mammalian cells is primarily regulated by the membrane-bound nucleoside transporters [1, 2]. As part of the protein superfamily of solute carriers (SLCs), these transporters can be further characterized in the sodium-dependent concentrative nucleoside transporters (SLC28) and the sodium-independent equilibrative transporters (ENTs, SLC29) [3]. Of the four defined isoforms belonging to the subfamily of ENTs (ENT1, ENT2, ENT3 and ENT4), ENT1 is most prevalent and widely distributed amongst different cell types such as cardiovascular cells and various neuronal tissues in the central nervous system [4–6]. Exemplary of the physiological relevance of ENT1 is the recent finding that knockdown or inhibition of ENT1-mediated inosine reuptake was identified as a mechanism to promote brown adipocyte differentiation and therefore cardiometabolic health [7]. In addition to inosine, reuptake of its precursor adenosine is also ENT1-mediated, making the transporter an important regulator of extracellular adenosine concentrations [1]. As a result, inhibition of adenosine reuptake leads to altered downstream signaling of pathways implicated with adenosine receptors. Through this indirect effect on adenosine receptor signaling, multiple physiological events such as vasodilation and neurotransmission are associated with ENT1 function [2]. For instance, ENT1 inhibition, and thus modulation of adenosine homeostasis, is directly linked to mitigation of Tau pathologies, one of the major pathogenic hallmarks in Alzheimer’s disease [8]. Furthermore, as uptake of anticancer and antiviral drugs such as gemcitabine and ribavirin is mediated by hENT1, its expression levels are directly correlated to the efficacy of these nucleoside-derived therapies [9, 10]. In short, due to its involvement in the transport of nucleosides and closely related analogues, ENT1 inhibition is a relevant therapeutic strategy to combat various pathophysiological conditions.

**Fig. 1:**
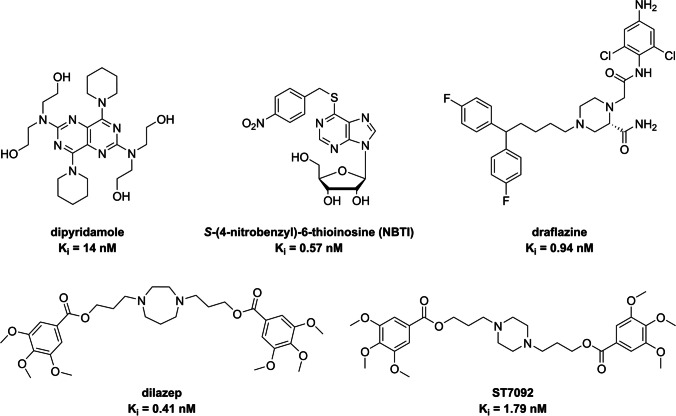
Chemical structures of reported hENT1 (therapeutic) inhibitors dipyridamole and dilazep, and molecular tools NBTI, draflazine and ST7092 and their corresponding K_i_ values. K_i_ values are as previously described, with the exception of ST7092 (data unpublished) [11]

In the context of cardiac implications like ischemia and hypertension, direct inhibition of ENT1 to increase extracellular adenosine levels has been studied as a therapeutic strategy [5]. To this end, multiple efforts have been made to develop adenosine reuptake inhibitors, resulting in structure–activity relationships of various chemically diverse molecules like NBTI, draflazine and the marketed antithrombotic agents dipyridamole and dilazep (Fig. 1) [12]. In addition to classical characterization of the inhibitory potency, kinetic profiles have been established [11, 13] and multiple ENT1 molecular tools (fluorescent [14] and photoaffinity probes [15]) have been developed to study the function and structure of the transporter. With the use of X-ray crystallography, two hENT1 crystal structures were elucidated using a biochemically stable variant of the protein in complex with nucleoside-derived inhibitor NBTI and non-nucleoside inhibitor dilazep (Fig. 1) [16]. Dilazep, a symmetrical cycloalkyldiamine, which is clinically used as a vasodilator, is structurally different compared to the well investigated scaffolds such as NBTI and dipyridamole. In spite of the aforementioned crystal structure and earlier efforts investigating dilazep derivatives [17] and close analogues hexobendine and ST7092 (Fig. 1) [18], little is known about the structure–activity relationship or the binding mechanisms of these non-nucleoside ENT1 inhibitors. In this study, we report the synthesis and pharmacological evaluation of 39 new dilazep-like derivatives based on the structure of ST7092 (Fig. 1) to explore the chemical diversity tolerated in the hENT1 binding pocket. 17 of these derivatives were equipped with an electrophilic warhead to attempt irreversible interaction with amino acid residue C439 of the binding pocket. Additionally, predicted binding modes of several inhibitors were examined with the use of molecular docking to gain insight into the different binding profiles characterized by [^3^H]NBTI displacement assays. The binding affinity characterization and predicted binding mode investigation of these new non-nucleoside hENT1 inhibitors may contribute to a better understanding of the hENT1 binding pocket and will aid in the further development of non-nucleoside-derived molecular tools to study the transporters’ function and binding mechanisms.

## Materials and methods

### Chemistry

#### General chemistry

All solvents and reagents were purchased from commercial sources and were of analytical grade. Demineralized water is referred to as H_2_O, as was used in all cases unless noted otherwise (e.g., brine). All reactions were routinely monitored with thin-layer chromatography (TLC), using aluminum silica gel coated 60 F_254_ plates from Merck (Darmstadt, Germany) and visualized by UV irradiation at 254 nm or staining with ninhydrin or KMnO_4_ solution. Purification by flash column chromatography was carried out with the use of silica gel irregular ZEOprep® particles (60–200 µm) from VWR (Amsterdam, The Netherlands) or using an Isolera™ One or Selekt automatic flash chromatography system from Biotage® (Uppsala, Sweden) with pre-packed cartridges (Phenomenex (Torrance, CA, USA) Gemini® Claricep™ (silica) or Biotage® Sfär C18 D Duo 100 Å 30 µm (C18)). Solutions were concentrated using a Heidolph (Schwabach, Germany) Hei-VAP Value rotary evaporator. Nuclear magnetic resonance (NMR) spectra were recorded on a Bruker (Billerica, MA, USA) AV-400 liquid spectrometer (^1^H NMR, 400 MHz and ^13^C NMR, 101 MHz) at ambient temperature and subsequently analyzed with MestReNova v14.1.0 software (Mestrelab Research S.L., Santiago de Compostela, Spain). Chemical shifts are reported in parts per million (ppm), designated by δ and corrected to the internal standard tetramethylsilane (δ = 0). Multiplicities are indicated by s, singlet; d, doublet; dd, doublet of doublets; ddd, doublet of doublet of doublets; td, triplet of doublets; t, triplet; dt, doublet of triplets; tt, triplet of triplets; q, quartet; p, pentet; m, multiplet; br s, broad singlet; br t, broad triplet. Coupling-constants (*J*) are reported in Hz. Mass and compound purity analyses were performed with liquid chromatography-mass spectrometry (LC–MS) using a LCMS-2020 system from Shimadzu (Kyoto, Japan) coupled to a Phenomenex Gemini® C18 110 Å column (50 mm × 3 mm × 3 μm). Samples were prepared by dissolving 0.3–0.8 mg of compound in 1 mL of a 1:1:1 mixture of CH_3_CN/H_2_O/tBuOH and were eluted using an isocratic system of H_2_O/CH_3_CN with 0.1% FA, using gradients from 100:0 to 60:40 and 90:10 to 10:90 in an elution time of 15 min. All tested compounds were determined to be of > 95% purity determined by HPLC.

#### Synthetic procedures

##### General procedure A:

To a stirred solution of the appropriate commercially available methylaminobenzoic acid (453 mg, 3.00 mmol, 1.0 equiv) in 1,4-dioxane (7.5 mL), was added dropwise Fmoc-Cl (0.72 mL, 3.60 mmol, 1.2 equiv). The mixture stirred for 10 min at 0 °C under N_2_ atmosphere followed by the addition of a suspension of K_2_CO_3_ (1.66 g, 12.0 mmol, 4.0 equiv) in 1,4-dioxane (7.5 mL) and the reaction mixture was allowed to stir at rt for 41 h. Subsequently, the mixture was diluted with H_2_O and neutralized to pH 7 with 0.5 M aqueous HCl. The aqueous phase was extracted with DCM (2 × 100 mL) and the combined organic phase was dried over MgSO_4_, filtrated and concentrated *in vacuo*. Flash column chromatography on silica gel using a gradient of 2 to 4% MeOH in DCM as mobile phase provided intermediates **1a** and **1b**.

##### General procedure B:

To a stirred solution of commercially available substituted benzoyl chloride (1.2 equiv) in DCM or 1,4-dioxane (0.2 M) at 0 °C under N_2_ atmosphere was added dropwise the appropriate bromoalcohol (1.0 equiv) and Et_3_N (2.0 equiv). The reaction mixture stirred for 0.5 h at 0 °C and overnight at rt. Subsequently, the mixture was quenched with H_2_O, diluted with DCM and the phases were separated. The aqueous phase was extracted with DCM (two times) and the combined organic phase was washed with brine, dried over MgSO_4_, filtrated and concentrated *in vacuo*. Flash column chromatography on silica gel using a gradient of EtOAc in PE as mobile phase provided intermediates **2a**-**2b**, **2d**-**2f**, **2h**, **2k**-**2l** and **2p**.

##### General procedure C:

A stirred mixture of the appropriate substituted benzoic acid (1.2 equiv) and thionyl chloride (0.2 M) was refluxed at 75 °C for 4 h under N_2_ atmosphere. After cooling down to rt the thionyl chloride was evaporated *in vacuo* and the crude benzoyl chloride was used as described in general procedure B to provide intermediates **2c**, **2g**, **2i**-**2j**, **2m** and** 8a**-**8e**.

##### General procedure D:

To a stirred solution of the appropriate substituted benzoic acid (1.0 equiv) in DCM (0.2 M) was added DMAP (cat.) and EDC·HCl (2.0 equiv). The mixture stirred for 1.5 h at rt before 3-bromopropan-1-ol (1.2 equiv) was added and the reaction continued at rt overnight under N_2_ atmosphere. The mixture was diluted with DCM and washed with H_2_O (two times). The aqueous phase was extracted with DCM and the combined organic phase was dried over MgSO_4_, filtrated and concentrated *in vacuo*. Flash column chromatography on silica gel using a gradient of EtOAc in PE as mobile phase provided intermediates **2n**-**2o** and **12a**-**12b**.

##### General procedure E:

To a stirred solution of the appropriate bromopropyl benzoate (1.0 equiv) in anhydrous DMF (0.2 M) were added 1-Boc-piperazine (1.2 equiv) and K_2_CO_3_ (2.0 equiv). The reaction mixture was stirred for 72 h at room temperature (rt) under N_2_ atmosphere followed by addition of H_2_O and EtOAc. After separation, the organic phase was washed four times with H_2_O, once with brine, dried over MgSO_4_, filtrated and concentrated *in vacuo*. Flash column chromatography on silica gel using a gradient of MeOH in DCM as mobile phase provided intermediates **3a**-**3c**. After NMR and LC–MS analyses, the formed intermediate was dissolved in DCM (0.2 M) and allowed to cool down to 0 °C after which TFA (20 equiv) was added dropwise. The mixture was stirred for 4 h, concentrated *in vacuo* and co-evaporated with toluene to remove excess TFA. The obtained oil was dissolved in EtOAc and subsequently co-evaporated with 4 N HCl in 1,4-dioxane to obtain intermediates **4a**-**4c** as dihydrochloride salts.

##### General procedure F:

The appropriate bromopropyl benzoate (1.0 equiv), appropriate intermediate **4** (1.2 equiv) and K_2_CO_3_ (4.0 equiv) were dissolved in anhydrous DMF (0.2 M). The reaction mixture stirred for 68 h at rt under N_2_ atmosphere followed by 4 h at 50 °C. EtOAc and H_2_O were added and after separation the organic phase was washed four times with H_2_O, once with brine. The organic phase was dried over MgSO_4_, filtrated and concentrated *in vacuo*. Flash column chromatography on silica gel using a gradient of MeOH in DCM as mobile phase provided final compounds **6a**-**6k**, **6u**-**6v**, **9a**, **9c**-**9f** and intermediates **9h**-**9m**, **13a**-**13b**.

##### General procedure G:

The appropriate bromopropyl benzoate (3.0 equiv), piperazine (1.0 equiv) and K_2_CO_3_ (4.0 equiv) were dissolved in anhydrous DMF (0.2 M). The reaction mixture stirred for 68 h at rt under N_2_ atmosphere followed by 4 h at 50 °C. EtOAc and H_2_O were added and after separation the organic phase was washed four times with H_2_O, once with brine. The organic phase was dried over MgSO_4_, filtrated and concentrated *in vacuo*. Flash column chromatography on silica gel using a gradient of MeOH in DCM as mobile phase provided final compounds **6p**-**6t**.

##### General procedure H:

To a stirred solution of intermediate **5** (1.0 equiv) in DCM (0.2 M) at 0 °C was added dropwise the commercially available substituted benzoyl chloride (1.1 equiv) and Et_3_N (3.0 equiv). The reaction mixture stirred for 1 h gradually warming up to rt under N_2_ atmosphere. Subsequently, the mixture was quenched with H_2_O, diluted with DCM and the phases were separated. The aqueous phase was extracted with DCM (two times) and the combined organic phase was washed with brine, dried over MgSO_4_, filtrated and concentrated *in vacuo*. Flash column chromatography on silica gel using a gradient of MeOH in DCM as mobile phase provided final compounds **6l** and **6m**.

##### General procedure I:

A stirred mixture of the appropriate substituted benzoic acid (1.2 equiv) and thionyl chloride (0.2 M) was refluxed at 75 °C for 4 h under N_2_ atmosphere. After cooling down to rt the thionyl chloride was evaporated *in vacuo* and the crude benzoyl chloride was used as described in general procedure H to provide final compounds **6n**, **6o** and **9b**.

##### General procedure J:

To a stirred solution of the appropriate commercially available chlorosulfonylbenzoic acid (1.0 equiv) in 1,4-dioxane (0.4 M) was added dropwise an aqueous solution of KHF_2_ (3.0 equiv, 2 M). The mixture was stirred at rt for 1 h followed by dilution with EtOAc. The organic phase was washed with H_2_O, dried over MgSO_4_, filtrated and concentrated *in vacuo* to provide intermediates **7a** and **7b**.

##### General procedure K:

To a stirred mixture of the appropriate commercially available amino-substituted benzoic acid (1.0 equiv) in 1,4-dioxane and H_2_O (1:1 or 2:1) was added Et_3_N or NaOH (2.0 equiv) followed by slow addition of Boc_2_O (2.0 equiv). The reaction mixture was stirred for 17 h at rt and subsequently acidified by addition of 1 M aqueous HCl until no further precipitation was observed. The formed suspension was filtrated, the residue rinsed with H_2_O and subsequently dissolved in EtOH followed by concentration *in vacuo* to give intermediates **7d-7h**.

##### General procedure L:

A stirred mixture of the appropriate substituted benzoic acid (1.2 equiv) in DCM or toluene (0.2 M) was allowed to cool down to 0 °C. Subsequently, oxalyl chloride (2.0 equiv) and two drops of DMF (cat.) were added and the reaction mixture was stirred for 4 h at 0 °C under N_2_ atmosphere. The solvent was evaporated *in vacuo* and the crude benzoyl chloride was used as described in general procedure B to provide intermediates **8f**-**8k**.

##### General procedure M:

To a stirred solution of 4-hydroxy-3,5-dimethoxybenzoic acid (1.0 equiv) in THF (0.2 M) at 0 °C was added tetrabutylammonium hydroxide (2.0 equiv) and the appropriate bromoalkylamine (1.5 equiv). The reaction mixture was stirred for two days under N_2_ atmosphere followed by concentration *in vacuo*. The obtained residue was dissolved in water and 4 M aqueous HCl was added until pH 4 was reached. The aqueous solution was extracted twice with DCM. The combined organic phase was dried over MgSO_4_, filtrated and concentrated *in vacuo*. Flash column chromatography on silica gel using a gradient of MeOH in DCM as mobile phase provided intermediates **11a** and **11b**.

##### General procedure N:

The appropriate Boc-protected intermediate was dissolved in DCM (0.2 M) and allowed to cool down to 0 °C after which TFA (20 equiv) was added dropwise. The mixture was stirred for 4 h, concentrated *in vacuo* and co-evaporated with toluene to remove the excess TFA. The obtained oil was dissolved in DCM (0.2 M) and the stirred solution was cooled to 0 °C. Acryloyl chloride (1.5 equiv) was added followed by Et_3_N (3.0 equiv), and the reaction mixture stirred for 2 h at 0 °C under N_2_ atmosphere. The mixture was diluted with DCM and subsequently washed twice with brine. The organic phase was dried over MgSO_4_, filtrated and concentrated *in vacuo*. Flash column chromatography on silica gel using a gradient of MeOH in DCM as mobile phase provided final compounds **10a-10f** and **14a-14b**.

**3-((((9H-fluoren-9-yl)methoxy)carbonyl)(methyl)amino)benzoic acid (1a)**. Intermediate **1a** was obtained from 3-(methylamino)benzoic acid as a white solid (312 mg, 0.83 mmol, 28%) following general procedure A. ^1^H NMR (400 MHz, CDCl_3_) δ 10.59 (br s, 1H), 8.04 – 7.93 (m, 2H), 7.68 (d, *J* = 7.6 Hz, 2H), 7.45 – 6.90 (m, 8H), 4.46 (d, *J* = 6.7 Hz, 2H), 4.12 (s, 1H), 3.31 (s, 3H). LC–MS (ESI +) *m/z* calcd. for C_23_H_19_NO_4_ [(M + H)]^+^: 374.14; found: 374.10. HPLC t_*R*_: 10.989 min.

**4-((((9H-fluoren-9-yl)methoxy)carbonyl)(methyl)amino)benzoic acid (1b).** Intermediate **1b** was obtained from 4-(methylamino)benzoic acid as a white solid (428 mg, 1.15 mmol, 38%) following general procedure A. ^1^H NMR (400 MHz, CDCl_3_) δ 8.15 – 8.01 (m, 2H), 7.80 – 7.69 (m, 2H), 7.48 – 7.33 (m, 4H), 7.30 – 7.11 (m, 4H), 4.53 (d, *J* = 6.3 Hz, 2H), 4.18 (t, *J* = 6.4 Hz, 1H), 3.32 (s, 3H). LC–MS (ESI +) *m/z* calcd. for C_23_H_19_NO_4_ [(M + H)]^+^: 374.14; found: 374.10. HPLC *t*_R_: 10.922 min.

**3-bromopropyl benzoate (2a).** Intermediate **2a** was obtained from benzoyl chloride following general procedure B. Column chromatography with 3% EtOAc in PE as mobile phase. Transparent oil (520 mg, 2.14 mmol, 86%). ^1^H NMR (400 MHz, CDCl_3_) δ 8.04 – 7.99 (m, 2H), 7.55 – 7.48 (m, 1H), 7.43 – 7.36 (m, 2H), 4.41 (t, *J* = 6.1 Hz, 2H), 3.50 (t, *J* = 6.6 Hz, 2H), 2.25 (p, *J* = 6.3 Hz, 2H). LC–MS (ESI +) *m/z* calcd. for C_10_H_11_BrO_2_ [(M + H)]^+^: 243.00; found: 242.90. HPLC *t*_R_: 10.765 min.

**3-bromopropyl 4-methylbenzoate (2b).** Intermediate **2b** was obtained from 4-methylbenzoyl chloride following general procedure B. Column chromatography with 0 to 4% EtOAc in PE as mobile phase. Transparent oil (556 mg, 2.16 mmol, 86%). ^1^H NMR (400 MHz, CDCl_3_) δ 7.96 – 7.90 (m, 2H), 7.26 – 7.21 (m, 2H), 4.45 (t, *J* = 6.0 Hz, 2H), 3.55 (t, *J* = 6.6 Hz, 2H), 2.41 (s, 3H), 2.31 (p, *J* = 6.6, 6.2 Hz, 2H). LC–MS (ESI +) *m/z* calcd. for C_11_H_13_BrO_2_ [(M + H)]^+^: 257.02; found: 256.95. HPLC *t*_R_: 11.284 min.

**3-bromopropyl 3,4-dimethybenzoate (2c).** Intermediate **2c** was obtained from 3,4-dimethybenzoic acid following general procedure C. Column chromatography with 20 to 50% DCM in PE as mobile phase. Transparent oil (513 mg, 1.89 mmol, 76%). ^1^H NMR (400 MHz, CDCl_3_) δ 7.78 (d, *J* = 1.9 Hz, 1H), 7.75 (dd, *J* = 7.9, 1.9 Hz, 1H), 7.16 (d, *J* = 7.8 Hz, 1H), 4.41 (t, *J* = 6.0 Hz, 2H), 3.53 (t, *J* = 6.6 Hz, 2H), 2.33 – 2.23 (m, 8H). LC–MS (ESI +) *m/z* calcd. for C_12_H_15_BrO_2_ [(M + H)]^+^: 271.03; found: 270.95. HPLC *t*_R_: 11.974 min.

**3-bromopropyl 3,5-dimethylbenzoate (2d).** Intermediate **2d** was obtained from 3,5-dimethylbenzoyl chloride following general procedure B. Column chromatography with 0 to 5% EtOAc in PE as mobile phase. Transparent oil (871 mg, 3.21 mmol, quant.). ^1^H NMR (400 MHz, CDCl_3_) δ 7.67 – 7.60 (m, 2H), 7.17 – 7.11 (m, 1H), 4.41 (t, *J* = 6.0 Hz, 2H), 3.52 (t, *J* = 6.6 Hz, 2H), 2.38 – 2.31 (m, 6H), 2.27 (p, *J* = 6.3 Hz, 2H). LC–MS (ESI +) *m/z* calcd. for C_12_H_15_BrO_7_ [(M + H)]^+^: 271.03; found: 270.95. HPLC *t*_R_: 12.128 min.

**3-bromopropyl 4-chlorobenzoate (2e).** Intermediate **2e** was obtained from 4-chlorobenzoyl chloride following general procedure B. Column chromatography with 2 to 5% EtOAc in PE as mobile phase. Transparent oil (765 mg, 2.75 mmol, quant.). ^1^H NMR (400 MHz, CDCl_3_) δ 7.95 (d, *J* = 8.6 Hz, 2H), 7.39 (d, *J* = 8.6 Hz, 2H), 4.45 (t, *J* = 6.1 Hz, 2H), 3.54 (t, *J* = 6.5 Hz, 2H), 2.31 (p, *J* = 6.3 Hz, 2H). LC–MS (ESI +) *m/z* calcd. for C_10_H_10_BrClO_2_ [(M + H)]^+^: 276.96; found: 276.85. HPLC *t*_R_: 11.559 min.

**3-bromopropyl 3,4-dichlorobenzoate (2f).** Intermediate **2f** was obtained from 3,4-dichlorobenzoyl chloride following general procedure B. Column chromatography with 2 to 4% EtOAc in PE as mobile phase. Yellow oil (703 mg, 2.25 mmol, 90%). ^1^H NMR (400 MHz, CDCl_3_) δ 8.10 (d, *J* = 2.0 Hz, 1H), 7.86 (dd, *J* = 8.4, 2.0 Hz, 1H), 7.53 (d, *J* = 8.3 Hz, 1H), 4.48 (t, *J* = 6.1 Hz, 2H), 3.54 (t, *J* = 6.5 Hz, 2H), 2.33 (p, *J* = 6.4 Hz, 2H).

**3-bromopropyl 3-(dimethylamino)benzoate (2g).** Intermediate **2g** was obtained from 3-(dimethylamino)benzoic acid following general procedure C. Column chromatography with 20 to 50% DCM in PE as mobile phase. Transparent oil (211 mg, 0.74 mmol, 21%). ^1^H NMR (400 MHz, CDCl_3_) δ 7.42 – 7.34 (m, 2H), 7.28 (t, *J* = 7.9 Hz, 1H), 6.91 (ddd, *J* = 8.3, 2.8, 1.1 Hz, 1H), 4.45 (t, *J* = 6.0 Hz, 2H), 3.54 (t, *J* = 6.6 Hz, 2H), 2.98 (s, 6H), 2.31 (p, *J* = 6.4 Hz, 2H). LC–MS (ESI +) *m/z* calcd. for C_12_H_16_BrNO_2_ [(M + H)]^+^: 286.04; found: 285.95. HPLC *t*_R_: 10.793 min.

**3-bromopropyl 4-(dimethylamino)benzoate (2h).** Intermediate **2h** was obtained from 4-(dimethylamino)benzoyl chloride following general procedure B. Column chromatography with 5 to 25% EtOAc in PE as mobile phase. Yellow oil (488 mg, 1.71 mmol, 68%). ^1^H NMR (400 MHz, CDCl_3_) δ 7.89 (d, *J* = 9.1 Hz, 2H), 6.62 (d, *J* = 9.1 Hz, 2H), 4.38 (t, *J* = 6.0 Hz, 2H), 3.53 (t, *J* = 6.7 Hz, 2H), 3.01 (s, 6H), 2.27 (p, *J* = 6.3 Hz, 2H). LC–MS (ESI +) *m/z* calcd. for C_12_H_16_BrNO_2_ [(M + H)]^+^: 286.04; found: 285.90. HPLC *t*_R_: 11.085 min.

**3-bromopropyl 3-((((9H-fluoren-9-yl)methoxy)carbonyl)(methyl)amino)benzoate (2i).** Intermediate **2i** was obtained from **1a** following general procedure C. Column chromatography with 10 to 25% EtOAc in PE as mobile phase. Yellow oil (297 mg, 0.71 mmol, 84%). ^1^H NMR (400 MHz, CDCl_3_) δ 7.95 – 7.86 (m, 2H), 7.69 (d, *J* = 7.6 Hz, 2H), 7.48 – 7.04 (m, 8H), 4.53 – 4.34 (m, 4H), 4.12 (s, 1H), 3.50 (t, *J* = 6.5 Hz, 2H), 3.30 (s, 3H), 2.29 (p, *J* = 6.3 Hz, 2H). LC–MS (ESI +) *m/z* calcd. for C_26_H_24_BrNO_4_ [(M + H)]^+^: 494.10; found: 494.10. HPLC *t*_R_: 12.951 min.

**3-bromopropyl 4-((((9H-fluoren-9-yl)methoxy)carbonyl)(methyl)amino)benzoate (2j).** Intermediate **2j** was obtained from **1b** following general procedure C. Column chromatography with 0 to 25% EtOAc in PE as mobile phase. Yellow oil (422 mg, 0.85 mmol, 29%). ^1^H NMR (400 MHz, CDCl_3_) δ 8.01 – 7.95 (m, 2H), 7.76 – 7.69 (m, 2H), 7.46 – 7.33 (m, 4H), 7.28 – 7.16 (m, 4H), 4.61 – 4.46 (m, 4H), 4.16 (t, *J* = 6.5 Hz, 1H), 3.57 (t, *J* = 6.5 Hz, 2H), 3.30 (s, 3H), 2.34 (p, *J* = 6.3 Hz, 2H). LC–MS (ESI +) *m/z* calcd. for C_26_H_24_BrNO_4_ [(M + H)]^+^: 494.10; found: 494.10. HPLC *t*_R_: 12.978 min.

**3-bromopropyl 2-methoxybenzoate (2k).** Intermediate **2k** was obtained from 2-methoxybenzoyl chloride following general procedure B. Column chromatography with 5 to 15% EtOAc in PE as mobile phase. Transparent oil (652 mg, 2.39 mmol, 95%). ^1^H NMR (400 MHz, CDCl_3_) δ 7.77 (dd, *J* = 8.0, 1.8 Hz, 1H), 7.45 (ddd, *J* = 7.9, 7.1, 1.8 Hz, 1H), 7.01 – 6.91 (m, 2H), 4.41 (t, *J* = 6.0 Hz, 2H), 3.86 (s, 3H), 3.55 (t, *J* = 6.6 Hz, 2H), 2.26 (p, *J* = 6.3 Hz, 2H). LC–MS (ESI +) *m/z* calcd. for C_11_H_13_BrO_3_ [(M + H)]^+^: 273.01; found: 272.90. HPLC *t*_R_: 10.530 min.

**3-bromopropyl 3-methoxybenzoate (2l).** Intermediate **2l** was obtained from 3-methoxybenzoyl chloride following general procedure B. Column chromatography with 5 to 15% EtOAc in PE as mobile phase. Transparent oil (1.27 g, 4.66 mmol, 78%). ^1^H NMR (400 MHz, CDCl_3_) δ 7.63 – 7.60 (m, 1H), 7.54 (dd, *J* = 2.8, 1.5 Hz, 1H), 7.33 (t, *J* = 8.0 Hz, 1H), 7.09 (ddd, *J* = 8.2, 2.7, 1.0 Hz, 1H), 4.44 (t, *J* = 6.1 Hz, 2H), 3.83 (s, 3H), 3.53 (t, *J* = 6.6 Hz, 2H), 2.30 (p, *J* = 6.3 Hz, 2H). LC–MS (ESI +) *m/z* calcd. for C_11_H_13_BrO_3_ [(M + H)]^+^: 273.01; found: 272.90. HPLC *t*_R_: 11.110 min.

**3-bromopropyl 4-methoxybenzoate (2m).** Intermediate **2m** was obtained from 4-methoxybenzoic acid following general procedure C. Column chromatography with 2 to 8% EtOAc in PE as mobile phase. Yellow oil (1.15 g, 4.21 mmol, 84%). ^1^H NMR (400 MHz, CDCl_3_) δ 8.02 – 7.96 (m, 2H), 6.95 – 6.89 (m, 2H), 4.44 (t, *J* = 6.0 Hz, 2H), 3.87 (s, 3H), 3.55 (t, *J* = 6.6 Hz, 2H), 2.31 (p, *J* = 6.2 Hz, 2H). LC–MS (ESI +) *m/z* calcd. for C_11_H_13_BrO_3_ [(M + H)]^+^: 273.01; found: 272.95. HPLC *t*_R_: 10.938 min.

**3-bromopropyl 3,4-dimethoxybenzoate (2n).** Intermediate **2n** was obtained from 3,4-dimethoxybenzoic acid following general procedure D. Column chromatography with 10 to 20% EtOAc in PE as mobile phase. Transparent oil (513 mg, 1.69 mmol, 28%). ^1^H NMR (400 MHz, CDCl_3_) δ 7.68 (dd, *J* = 8.4, 2.0 Hz, 1H), 7.54 (d, *J* = 2.0 Hz, 1H), 6.89 (d, *J* = 8.4 Hz, 1H), 4.45 (t, *J* = 6.0 Hz, 2H), 3.94 (s, 3H), 3.94 (s, 3H), 3.55 (t, *J* = 6.5 Hz, 2H), 2.32 (p, *J* = 6.3 Hz, 2H). LC–MS (ESI +) *m/z* calcd. for C_12_H_15_BrO_4_ [(M + H)]^+^: 303.02; found: 302.95. HPLC *t*_R_: 10.466 min.

**3-bromopropyl 3,5-dimethoxybenzoate (2o)** Intermediate **2o** was obtained from 3,5-dimethoxybenzoic acid following general procedure D. Column chromatography with 40 to 70% DCM in PE as mobile phase. Transparent oil (1.09 g, 3.60 mmol, 60%). ^1^H NMR (400 MHz, CDCl_3_) δ 7.16 (d, *J* = 2.4 Hz, 2H), 6.64 (t, *J* = 2.4 Hz, 1H), 4.45 (t, *J* = 6.0 Hz, 2H), 3.82 (s, 6H), 3.54 (t, *J* = 6.5 Hz, 2H), 2.31 (p, *J* = 6.3 Hz, 2H). LC–MS (ESI +) *m/z* calcd. for C_12_H_15_BrO_4_ [(M + H)]^+^: 303.02; found: 302.95. HPLC *t*_R_: 11.275 min.

**3-bromopropyl 3,4,5-trimethoxybenzoate (2p).** Intermediate **2p** was obtained from 3,4,5-trimethoxybenzoyl chloride following general procedure B. Column chromatography with 5 to 15% EtOAc in PE as mobile phase. White solid (6.66 g, 18.2 mmol, 91%). ^1^H NMR (400 MHz, CDCl_3_) δ 7.29 (s, 2H), 4.47 (t, *J* = 6.1 Hz, 2H), 3.92 (s, 9H), 3.54 (t, *J* = 6.5 Hz, 2H), 2.34 (p, *J* = 6.3 Hz, 2H). LC–MS (ESI +) *m/z* calcd. for C_13_H_17_BrO_5_ [(M + H)]^+^: 333.03; found: 333.00. HPLC *t*_R_: 10.538 min.

**tert-butyl 4-(3-((2-methoxybenzoyl)oxy)propyl)piperazine-1-carboxylate (3a).** Intermediates **3a** and **4a** were obtained from **2k** following general procedure E. Column chromatography with 0 to 1.5% MeOH in DCM as mobile phase. Transparent oil (275 mg, 0.73 mmol, 53%). ^1^H NMR (400 MHz, CDCl_3_) δ 7.78 (dd, *J* = 7.9, 1.8 Hz, 1H), 7.47 (ddd, *J* = 8.4, 7.4, 1.8 Hz, 1H), 7.02 – 6.95 (m, 2H), 4.36 (t, *J* = 6.4 Hz, 2H), 3.90 (s, 3H), 3.44 (t, *J* = 4.7 Hz, 4H), 2.51 (t, *J* = 7.5, 7.1 Hz, 2H), 2.41 (t, *J* = 5.1 Hz, 4H), 1.99 – 1.90 (m, 2H), 1.46 (s, 9H). LC–MS (ESI +) *m/z* calcd. for C_20_H_30_N_2_O_5_ [(M + H)]^+^: 379.22; found: 379.20. HPLC *t*_R_: 6.521 min. **3-(piperazin-1-yl)propyl 2-methoxybenzoate dihydrochloride (4a).** White solid (quant.) used without further analyses.

**tert-butyl 4-(3-((3,5-dimethoxybenzoyl)oxy)propyl)piperazine-1-carboxylate (3b).** Intermediates **3b** and **4b** were obtained from **2o** following general procedure E. Column chromatography with 1 to 3% MeOH in DCM as mobile phase. Transparent oil (1.16 g, 2.85 mmol, 80%). ^1^H NMR (400 MHz, CDCl_3_) δ 7.18 (d, *J* = 2.4 Hz, 2H), 6.65 (t, *J* = 2.4 Hz, 1H), 4.37 (t, *J* = 6.5 Hz, 2H), 3.83 (s, 6H), 3.43 (t, *J* = 5.1 Hz, 4H), 2.50 (t, *J* = 7.1 Hz, 2H), 2.40 (t, *J* = 5.0 Hz, 4H), 2.02 – 1.90 (m, 2H), 1.46 (s, 9H). **3-(piperazin-1-yl)propyl 3,5-dimethoxybenzoate dihydrochloride (4b).** White solid (954 mg, 2.50 mmol, 88%) used without further analyses.

**tert-butyl 4-(3-((3,4,5-trimethoxybenzoyl)oxy)propyl)piperazine-1-carboxylate (3c).** Intermediates **3c** and **4c** were obtained from **2p** following general procedure E. Column chromatography with 2% MeOH in DCM as mobile phase. Transparent oil (4.95 g, 11.3 mmol, 88%). ^1^H NMR (400 MHz, CDCl_3_) δ 7.30 (s, 2H), 4.39 (t, *J* = 6.6, 2.1 Hz, 2H), 3.92 (s, 9H), 3.52 – 3.34 (m, 4H), 2.51 (t, *J* = 7.0 Hz, 2H), 2.47 – 2.33 (m, 4H), 1.98 (p, *J* = 6.7, 6.3 Hz, 2H), 1.47 (s, 9H). LC–MS (ESI +) *m/z* calcd. for C_22_H_34_N_2_O_7_ [(M + H)]^+^: 439.24; found: 439.15. HPLC *t*_R_: 6.746 and 7.023 min. **3-(piperazin-1-yl)propyl 3,4,5-trimethoxybenzoate dihydrochloride (4c).** White solid (4.29 g, 10.4 mmol, 91%) used without further analyses.

**3-(4-(3-hydroxypropyl)piperazin-1-yl)propyl 3,4,5-trimethoxybenzoate (5).** To a stirred solution of intermediate **4c** (1.23 g, 3.00 mmol, 1.0 equiv) in anhydrous DMF (15.0 mL) was added 3-bromopropan-1-ol (0.29 mL, 3.30 mmol, 1.1 equiv) and K_2_CO_3_ (1.24 g, 9.00 mmol, 3.0 equiv). The reaction mixture was allowed to stir overnight at rt under N_2_ atmosphere followed by filtration and concentration *in vacuo*. The acquired oil was dissolved in EtOAc (50 mL) and subsequently washed with H_2_O (50 mL). The aqueous phase was extracted with EtOAc (50 mL) and the combined organic phases were washed with brine (50 mL), dried over MgSO_4_, filtrated and concentrated *in vacuo*. The crude product was purified using flash column chromatography on silica gel with 10% MeOH in DCM as mobile phase to obtain intermediate **5** (773 mg, 1.95 mmol, 65%) as a transparent oil. ^1^H NMR (400 MHz, MeOD) δ 7.32 (s, 2H), 4.36 (t, *J* = 6.4 Hz, 2H), 3.88 (s, 6H), 3.82 (s, 3H), 3.61 (t, *J* = 6.2 Hz, 2H), 2.60 – 2.39 (m, 12H), 2.03 – 1.94 (m, 2H), 1.79 – 1.68 (m, 2H). LC–MS (ESI +) *m/z* calcd. for C_20_H_32_N_2_O_6_ [(M + H)]^+^: 396.23; found: 397.20. HPLC *t*_R_: 0.818 min.

**3-(4-(3-(benzoyloxy)propyl)piperazin-1-yl)propyl 3,4,5-trimethoxybenzoate (6a).** Final compound **6a** was obtained from intermediates **2a** and **4c** following general procedure F. Column chromatography with 2 to 4.5% MeOH in DCM as mobile phase. Yellow oil (884 mg, 1.77 mmol, 83%). ^1^H NMR (400 MHz, CDCl_3_) δ 8.04 (d, *J* = 7.2 Hz, 2H), 7.55 (tt, *J* = 7.4, 1.6 Hz, 1H), 7.43 (t, *J* = 7.9 Hz, 2H), 7.30 (s, 2H), 4.38 (t, *J* = 6.5 Hz, 4H), 3.91 (s, 9H), 2.59 – 2.41 (m, 12H), 2.03 – 1.93 (m, 4H). ^13^C NMR (101 MHz, CDCl_3_) 166.4, 166.0, 152.8, 142.0, 132.7, 130.2, 129.4, 128.2, 125.2, 106.6, 63.4, 63.3, 60.7, 56.1, 55.0, 53.1, 53.1, 26.2, 26.1. LC–MS (ESI +) *m/z* calcd. for C_27_H_36_N_2_O_7_ [(M + H)]^+^: 501.26; found: 501.15. HPLC *t*_R_: 8.096 min.

**3-(4-(3-((4-methylbenzoyl)oxy)propyl)piperazin-1-yl)propyl 3,4,5-trimethoxybenzoate (6b)**. Final compound **6b** was obtained from intermediates **2b** and **4c** following general procedure F. Column chromatography with 2 to 4% MeOH in DCM as mobile phase. Yellow oil (272 mg, 0.53 mmol, 66%). ^1^H NMR (400 MHz, CDCl_3_) δ 7.92 (d, *J* = 8.2 Hz, 2H), 7.30 (s, 2H), 7.22 (d, *J* = 8.0 Hz, 2H), 4.45 – 4.29 (m, 4H), 3.90 (s, 9H), 2.57 – 2.45 (m, 12H), 2.38 (s, 3H), 2.03 – 1.89 (m, 4H). ^13^C NMR (101 MHz, CDCl_3_) δ 166.2, 165.8, 152.6, 143.2, 141.9, 129.3, 128.8, 127.3, 125.1, 106.5, 63.3, 62.9, 60.5, 55.9, 54.8, 52.9, 52.9, 26.0, 21.3. LC–MS (ESI +) *m/z* calcd. for C_28_H_38_N_2_O_7_ [(M + H)]^+^: 515.28; found: 515.35. HPLC *t*_R_: 7.544 min.

**3-(4-(3-((3,4-dimethylbenzoyl)oxy)propyl)piperazin-1-yl)propyl 3,4,5-trimethoxybenzoate (6c).** Final compound **6c** was obtained from intermediates **2c** and **4c** following general procedure F. Automatic column chromatography with 1 to 6% MeOH in DCM as mobile phase on Biotage Isolera One. Transparent oil (40.4 mg, 0.08 mmol, 14%). ^1^H NMR (400 MHz, CDCl_3_) δ 7.82 – 7.78 (m, 1H), 7.76 (dd, *J* = 7.8, 1.9 Hz, 1H), 7.29 (s, 2H), 7.19 (d, *J* = 7.9 Hz, 1H), 4.41 – 4.31 (m, 4H), 3.91 (s, 9H), 2.84 – 2.39 (m, 12H), 2.31 (s, 3H), 2.30 (s, 3H), 2.05 – 1.89 (m, 4H). ^13^C NMR (101 MHz, CDCl_3_) δ 167.0, 166.3, 153.0, 142.4, 136.8, 130.7, 129.8, 128.0, 127.2, 125.5, 106.9, 63.7, 63.3, 61.1, 56.4, 55.3, 55.2, 53.3, 53.2, 26.4, 26.4, 20.1, 19.8. LC–MS (ESI +) *m/z* calcd. for C_29_H_40_N_2_O_7_ [(M + H)]^+^: 529.29; found: 529.30. HPLC *t*_R_: 7.807 min.

**3-(4-(3-((3,5-dimethylbenzoyl)oxy)propyl)piperazin-1-yl)propyl 3,4,5-trimethoxybenzoate (6d).** Final compound **6d** was obtained from intermediates **2d** and **4c** following general procedure F. Automatic column chromatography with 2 to 6% MeOH in DCM as mobile phase on Biotage Isolera One. Yellow oil (241 mg, 0.46 mmol, 57%). ^1^H NMR (400 MHz, CDCl_3_) δ 7.68 – 7.62 (m, 2H), 7.30 (s, 2H), 7.20 – 7.14 (m, 1H), 4.44 – 4.30 (m, 4H), 3.91 (s, 9H), 2.86 – 2.36 (m, 12H), 2.36 (s, 6H), 2.04 – 1.89 (m, 4H). ^13^C NMR (101 MHz, CDCl_3_) δ 166.9, 166.2, 152.9, 142.2, 138.0, 134.5, 130.2, 127.2, 125.4, 106.8, 63.6, 63.3, 60.9, 56.2, 55.1, 55.1, 53.2, 53.2, 26.3, 21.2. LC–MS (ESI +) *m/z* calcd. for C_29_H_40_N_2_O_7_ [(M + H)]^+^: 528.29; found: 529.30. HPLC *t*_R_: 7.898 min.

**3-(4-(3-((4-chlorobenzoyl)oxy)propyl)piperazin-1-yl)propyl 3,4,5-trimethoxybenzoate (6e)**. Final compound **6e** was obtained from intermediates **2e** and **4c** following general procedure F. Automatic column chromatography with 0 to 6% MeOH in DCM as mobile phase on Biotage Isolera One. Yellow oil (306 mg, 0.57 mmol, 71%). ^1^H NMR (400 MHz, CDCl_3_) δ 7.97 (d, *J* = 8.6 Hz, 2H), 7.42 (d, *J* = 8.5 Hz, 2H), 7.29 (s, 2H), 4.37 (t, *J* = 6.5 Hz, 4H), 3.91 (s, 9H), 2.80 – 2.23 (m, 12H), 2.03 – 1.92 (m, 4H). ^13^C NMR (101 MHz, CDCl_3_) δ 166.4, 165.9, 153.1, 139.5, 131.1, 128.8, 125.5, 106.9, 63.8, 63.7, 61.1, 56.4, 55.3, 55.2, 53.3, 26.4, 26.4. LC–MS (ESI +) *m/z* calcd. for C_27_H_35_ClN_2_O_7_ [(M + H)]^+^: 535.22; found: 535.15. HPLC *t*_R_: 7.716 min.

**3-(4-(3-((3,4-dichlorobenzoyl)oxy)propyl)piperazin-1-yl)propyl 3,4,5-trimethoxybenzoate (6f).** Final compound **6f** was obtained from intermediates **2f** and **4c** following general procedure F. Automatic column chromatography with 2 to 6% MeOH in DCM as mobile phase on Biotage Isolera One. Orange oil (301 mg, 0.53 mmol, 66%). ^1^H NMR (400 MHz, CDCl_3_) δ 8.10 (d, *J* = 2.0 Hz, 1H), 7.85 (dd, *J* = 8.4, 2.0 Hz, 1H), 7.53 (d, *J* = 8.4 Hz, 1H), 7.29 (s, 2H), 4.42 – 4.34 (m, 4H), 3.91 (s, 9H), 2.80 – 2.31 (m, 12H), 2.03 – 1.92 (m, 4H). ^13^C NMR (101 MHz, CDCl_3_) δ 166.3, 164.9, 153.0, 142.3, 137.7, 133.0, 131.6, 130.7, 130.3, 128.8, 125.5, 106.9, 64.2, 63.7, 61.1, 56.4, 55.2, 55.1, 53.3, 26.4, 26.3. LC–MS (ESI +) *m/z* calcd. for C_27_H_34_Cl_2_N2O_7_ [(M + H)]^+^: 569.18; found: 569.10. HPLC *t*_R_: 8.162 min.

**3-(4-(3-((3-(dimethylamino)benzoyl)oxy)propyl)piperazin-1-yl)propyl 3,4,5-trimethoxybenzoate (6g).** Final compound **6g** was obtained from intermediates **2g** and **4c** following general procedure F. Column chromatography with 6% MeOH in EtOAc as mobile phase. Yellow oil (227 mg, 0.42 mmol, 58%). ^1^H NMR (400 MHz, CDCl_3_) δ 7.42 – 7.34 (m, 2H), 7.29 (s, 2H), 7.30 – 7.28 (m, 1H), 6.90 (ddd, *J* = 8.2, 2.8, 1.0 Hz, 1H), 4.44 – 4.28 (m, 4H), 3.91 (s, 9H), 2.99 (s, 6H), 2.78 – 2.13 (m, 12H), 2.04 – 1.87 (m, 4H). ^13^C NMR (101 MHz, CDCl_3_) δ 167.3, 166.3, 153.0, 150.5, 142.2, 131.0, 129.0, 125.4, 117.5, 116.8, 113.2, 106.8, 63.7, 63.3, 61.0, 56.3, 55.2, 55.2, 53.2, 53.2, 40.6, 26.3. LC–MS (ESI +) *m/z* calcd. for C_29_H_41_N_3_O_7_ [(M + H)]^+^: 544.30; found: 544.25. HPLC *t*_R_: 7.108 and 7.398 min.

**3-(4-(3-((4-(dimethylamino)benzoyl)oxy)propyl)piperazin-1-yl)propyl 3,4,5-trimethoxybenzoate (6h).** Final compound **6h** was obtained from intermediates **2h** and **4c** following general procedure F. Column chromatography with 1 to 3.5% MeOH in DCM as mobile phase. Transparent oil (103 mg, 0.19 mmol, 24%). ^1^H NMR (400 MHz, CDCl_3_) δ 7.90 (d, *J* = 9.1 Hz, 2H), 7.29 (s, 2H), 6.64 (d, *J* = 9.1 Hz, 2H), 4.37 (t, *J* = 6.5 Hz, 2H), 4.31 (t, *J* = 6.4 Hz, 2H), 3.91 (s, 6H), 3.91 (s, 3H), 3.04 (s, 6H), 2.81 – 2.25 (m, 12H), 2.04 – 1.88 (m, 4H). ^13^C NMR (101 MHz, CDCl_3_) δ 167.0, 166.3, 153.3, 153.0, 142.2, 131.3, 125.4, 117.1, 110.7, 106.8, 63.7, 62.6, 61.0, 56.3, 55.3, 55.2, 53.3, 53.2, 40.1, 26.5, 26.3. LC–MS (ESI +) *m/z* calcd. for C_29_H_41_N_3_O_7_ [(M + H)]^+^: 544.30; found: 544.20. HPLC *t*_R_: 7.617 min.

**3-(4-(3-((4-(methylamino)benzoyl)oxy)propyl)piperazin-1-yl)propyl 3,4,5-trimethoxybenzoate (6i).** Final compound **6i** was obtained from intermediates **2i** and **4c** following general procedure F. Column chromatography with 2 to 6% MeOH in DCM as mobile phase. Yellow oil (64.0 mg, 0.12 mmol, 48%). ^1^H NMR (400 MHz, CDCl_3_) δ 7.86 (d, *J* = 8.9 Hz, 2H), 7.29 (s, 2H), 6.55 (d, *J* = 9.0 Hz, 2H), 4.37 (t, *J* = 6.5 Hz, 2H), 4.30 (t, *J* = 6.4 Hz, 2H), 4.28 – 4.21 (m, 1H), 3.91 (s, 9H), 2.88 (d, *J* = 4.9 Hz, 3H), 2.83 – 2.21 (m, 12H), 2.05 – 1.87 (m, 4H). ^13^C NMR (101 MHz, CDCl_3_) δ 167.0, 166.3, 153.0, 153.0, 142.2, 131.6, 125.5, 118.4, 111.1, 106.8, 63.7, 62.7, 61.0, 56.3, 55.3, 55.2, 53.3, 53.3, 30.2, 26.5, 26.4. LC–MS (ESI +) *m/z* calcd. for C_28_H_39_N_3_O_7_ [(M + H)]^+^: 530.29; found: 530.25. HPLC *t*_R_: 9.641 min.

**3-(4-(3-((3-(methylamino)benzoyl)oxy)propyl)piperazin-1-yl)propyl 3,4,5-trimethoxybenzoate (6j).** Final compound **6j** was obtained from intermediates **2j** and **4c** following general procedure F. Column chromatography with 2 to 6% MeOH in DCM as mobile phase. Yellow oil (147 mg, 0.28 mmol, 46%). ^1^H NMR (400 MHz, CDCl_3_) δ 7.39 – 7.34 (m, 1H), 7.30 (s, 2H), 7.26 (dd, *J* = 2.5, 1.5 Hz, 1H), 7.23 (t, *J* = 7.9 Hz, 1H), 6.77 (ddd, *J* = 8.1, 2.6, 1.0 Hz, 1H), 4.46 – 4.28 (m, 4H), 3.91 (m, 10H), 2.86 (s, 3H), 2.78 – 2.24 (m, 12H), 2.03 – 1.90 (m, 4H). ^13^C NMR (101 MHz, CDCl_3_) δ 167.1, 166.2, 152.9, 149.3, 142.1, 131.2, 129.1, 125.4, 118.2, 116.8, 112.8, 106.7, 63.6, 63.3, 60.9, 56.2, 55.1, 55.1, 53.2, 53.2, 30.7, 26.3. LC–MS (ESI +) *m/z* calcd. for C_28_H_39_N_3_O_7_ [(M + H)]^+^: 530.29; found: 530.30. HPLC *t*_R_: 6.675 and 6.981 min.

**3-(4-(3-((2-methoxybenzoyl)oxy)propyl)piperazin-1-yl)propyl 3,4,5-trimethoxybenzoate (6k).** Final compound **6k** was obtained from intermediates **2k** and **4c** following general procedure F. Automatic column chromatography with 0 to 5% MeOH in DCM as mobile phase on Biotage Isolera One. Orange oil (230 mg, 0.43 mmol, 54%). ^1^H NMR (400 MHz, CDCl_3_) δ 7.78 (ddd, *J* = 8.0, 2.0, 0.8 Hz, 1H), 7.51 – 7.43 (m, 1H), 7.30 (s, 2H), 7.02 – 6.94 (m, 2H), 4.47 – 4.27 (m, 4H), 3.91 (s, 9H), 3.90 (s, 3H), 3.34 – 2.17 (m, 12H), 2.05 – 1.89 (m, 4H). ^13^C NMR (101 MHz, CDCl_3_) δ 166.3, 166.3, 159.2, 153.0, 142.2, 133.5, 131.6, 125.4, 120.3, 120.1, 112.0, 106.8, 63.6, 63.3, 61.0, 56.3, 56.0, 55.2, 55.2, 53.2, 53.2, 26.3, 26.3. LC–MS (ESI +) *m/z* calcd. for C_28_H_38_N_2_O_8_ [(M + H)]^+^: 531.27; found: 531.25. HPLC *t*_R_: 7.156 min.

**3-(4-(3-((3-methoxybenzoyl)oxy)propyl)piperazin-1-yl)propyl 3,4,5-trimethoxybenzoate (6l).** Final compound **6l** was obtained from intermediate **5** and 3-methoxybenzoyl chloride following general procedure H. Column chromatography with 3 to 6% MeOH in DCM as mobile phase. Transparent oil (36.6 mg, 0.07 mmol, 14%). ^1^H NMR (400 MHz, CDCl_3_) δ 7.63 (dt, *J* = 7.7, 1.2 Hz, 1H), 7.55 (dd, *J* = 2.8, 1.5 Hz, 1H), 7.35 (t, *J* = 8.0 Hz, 1H), 7.29 (s, 2H), 7.10 (ddd, *J* = 8.3, 2.7, 1.0 Hz, 1H), 4.37 (t, *J* = 6.3 Hz, 4H), 3.91 (s, 9H), 3.85 (s, 3H), 2.66 – 2.41 (m, 12H), 2.04 – 1.92 (m, 4H). ^13^C NMR (101 MHz, CDCl_3_) δ 166.6, 166.4, 159.7, 153.1, 142.3, 131.8, 129.5, 125.5, 122.1, 119.4, 114.2, 106.9, 63.7, 63.6, 61.1, 56.4, 55.6, 55.2, 53.3, 26.4, 26.4. LC–MS (ESI +) *m/z* calcd. for C_28_H_38_N_2_O_8_ [(M + H)]^+^: 531.27; found: 531.25. HPLC *t*_R_: 7.338 min.

**3-(4-(3-((4-methoxybenzoyl)oxy)propyl)piperazin-1-yl)propyl 3,4,5-trimethoxybenzoate (6m).** Final compound **6m** was obtained from intermediate **5** and 4-methoxybenzoyl chloride following general procedure H. Column chromatography with 3 to 6% MeOH in DCM as mobile phase. Transparent oil (285 mg, 0.47 mmol, 94%). ^1^H NMR (400 MHz, CDCl_3_) δ 7.99 (d, *J* = 9.1 Hz, 2H), 7.30 (s, 2H), 6.92 (d, *J* = 9.1 Hz, 2H), 4.37 (t, *J* = 6.5 Hz, 2H), 4.34 (t, *J* = 6.5 Hz, 2H), 3.91 (s, 6H), 3.90 (s, 3H), 3.85 (s, 3H), 2.69 – 2.48 (m, 12H), 2.06 – 1.90 (m, 4H). ^13^C NMR (101 MHz, CDCl_3_) δ 166.2, 166.1, 163.3, 152.9, 142.1, 131.5, 125.3, 122.6, 113.5, 106.7, 63.4, 62.9, 60.8, 56.2, 55.4, 55.02, 54.97, 52.90, 52.90, 26.13, 26.09. LC–MS (ESI +) *m/z* calcd. for C_28_H_38_N_2_O_8_ [(M + H)]^+^: 531.27; found 531.25. HPLC *t*_R_: 7.291 min.

**3-(4-(3-((3,4-dimethoxybenzoyl)oxy)propyl)piperazin-1-yl)propyl 3,4,5-trimethoxybenzoate (6n).** Final compound **6n** was obtained from intermediate **5** and 3,4-dimethoxybenzoic acid following general procedure I. Column chromatography with 3 to 6% MeOH in DCM as mobile phase. Transparent oil (36.9 mg, 0.07 mmol, 33%). ^1^H NMR (400 MHz, CDCl_3_) δ 7.67 (dd, *J* = 8.4, 2.0 Hz, 1H), 7.54 (d, *J* = 2.0 Hz, 1H), 7.29 (s, 2H), 6.89 (d, *J* = 8.5 Hz, 1H), 4.36 – 4.33 (m, 4H), 3.94 (s, 3H), 3.93 (s, 3H), 3.91 (s, 6H), 3.91 (s, 3H), 2.75 – 2.37 (m, 12H), 2.05 – 1.93 (m, 4H). ^13^C NMR (101 MHz, CDCl_3_) δ 166.5, 166.3, 153.1, 153.0, 148.7, 142.3, 125.4, 123.6, 122.9, 112.0, 110.3, 106.9, 63.7, 63.3, 61.0, 56.4, 56.1, 56.1, 55.23, 55.19, 53.2, 26.4, 26.3. LC–MS (ESI +) *m/z* calcd. for C_29_H_40_N_2_O_9_ [(M + H)]^+^: 561.28; found: 561.25. HPLC *t*_R_: 7.093 min.

**3-(4-(3-((3,5-dimethoxybenzoyl)oxy)propyl)piperazin-1-yl)propyl 3,4,5-trimethoxybenzoate (6o).** Final compound **6o** was obtained from intermediate **5** and 3,5-dimethoxybenzoic acid following general procedure I. Column chromatography with 3 to 6% MeOH in DCM as mobile phase. Transparent oil (82.0 mg, 0.15 mmol, 73%). ^1^H NMR (400 MHz, CDCl_3_) δ 7.29 (s, 2H), 7.18 (d, *J* = 2.4 Hz, 2H), 6.65 (t, *J* = 2.4 Hz, 1H), 4.37 (t, *J* = 6.3 Hz, 2H), 4.37 (t, *J* = 6.3 Hz, 2H), 3.91 (s, 6H), 3.91 (s, 3H), 3.83 (s, 6H), 2.70 – 2.39 (m, 12H), 2.05 – 1.92 (m, 2H). ^13^C NMR (101 MHz, CDCl_3_) δ 166.4, 166.3, 160.7, 153.0, 142.2, 132.3, 125.4, 107.2, 106.8, 105.5, 63.6, 61.0, 56.3, 55.6, 55.2, 55.1, 53.2, 53.2, 26.3, 26.3. LC–MS (ESI +) *m/z* calcd. for C_29_H_40_N_2_O_9_ [(M + H)]^+^: 561.28; found: 561.25. HPLC *t*_R_: 7.540 min.

**piperazine-1,4-diylbis(propane-3,1-diyl) bis(2-methoxybenzoate) (6p).** Final compound **6p** was obtained from intermediate **2k** following general procedure G. Column chromatography with 3 to 5% MeOH in DCM as mobile phase. Transparent oil (492 mg, 1.05 mmol, 47%). ^1^H NMR (400 MHz, CDCl_3_) δ 7.78 (dd, *J* = 7.9, 1.8 Hz, 2H), 7.49 – 7.41 (m, 2H), 6.99 – 6.92 (m, 4H), 4.34 (t, *J* = 6.4 Hz, 4H), 3.87 (s, 6H), 2.70–2.40 (m, 12H), 1.94 (p, *J* = 6.8 Hz, 4H). ^13^C NMR (101 MHz, CDCl_3_) δ 165.9, 158.8, 133.2, 131.2, 119.9, 119.8, 111.7, 62.9, 55.6, 54.8, 52.8, 25.9. LC–MS (ESI +) *m/z* calcd. for C_26_H_34_N_2_O_6_ [(M + H)]^+^: 471.25; found: 471.15. HPLC *t*_R_: 6.734 min.

**piperazine-1,4-diylbis(propane-3,1-diyl) bis(3-methoxybenzoate) (6q).** Final compound **6q** was obtained from intermediate **2l** following general procedure G. Column chromatography with 3 to 5% MeOH in DCM as mobile phase. Transparent oil (450 mg, 0.96 mmol, 45%). ^1^H NMR (400 MHz, CDCl_3_) δ 7.63 (dt, *J* = 7.6, 1.2 Hz, 2H), 7.55 (dd, *J* = 2.7, 1.5 Hz, 2H), 7.33 (t, *J* = 8.0 Hz, 2H), 7.08 (ddd, *J* = 8.2, 2.7, 1.0 Hz, 2H), 4.36 (t, *J* = 6.5 Hz, 4H), 3.83 (s, 6H), 2.60 – 2.41 (m, 12H), 1.96 (p, *J* = 7.1 Hz, 4H). ^13^C NMR (101 MHz, CDCl_3_) δ 166.2, 159.4, 131.5, 129.2, 121.7, 119.1, 114.0, 63.4, 55.2, 54.9, 53.0, 26.1. LC–MS (ESI +) *m/z* calcd. for C_26_H_34_N_2_O_6_ [(M + H)]^+^: 471.25; found: 471.25. HPLC *t*_R_: 7.020 and 7.365 min.

**piperazine-1,4-diylbis(propane-3,1-diyl) bis(4-methoxybenzoate) (6r).** Final compound **6r** was obtained from intermediate **2m** following general procedure G. Automatic column chromatography with 3 to 5% MeOH in DCM as mobile phase on Biotage Isolera One. Transparent oil (365 mg, 0.78 mmol, 55%). ^1^H NMR (400 MHz, CDCl_3_) 8.02 – 7.95 (m, 4H), 6.96 – 6.88 (m, 4H), 4.34 (t, *J* = 6.5 Hz, 4H), 3.85 (s, 6H), 2.64 – 2.31 (m, 12H), 2.02 – 1.85 (m, 4H). ^13^C NMR (101 MHz, CDCl_3_) δ 166.4, 163.4, 131.6, 122.8, 113.6, 63.2, 55.5, 55.2, 53.2, 26.4. LC–MS (ESI +) *m/z* calcd. for C_26_H_34_N_2_O_6_ [(M + H)]^+^: 471.25; found: 471.15. HPLC *t*_R_: 7.050 and 7.294 min.

**piperazine-1,4-diylbis(propane-3,1-diyl) bis(3,4-dimethoxybenzoate) (6s).** Final compound **6s** was obtained from intermediate **2n** following general procedure G. Column chromatography with 0.5 to 4% MeOH in DCM as mobile phase. Transparent oil (59.7 mg, 0.77 mmol, 15%). ^1^H NMR (400 MHz, CDCl_3_) δ 7.68 (dd, *J* = 8.4, 2.0 Hz, 2H), 7.54 (d, *J* = 2.0 Hz, 2H), 6.89 (d, *J* = 8.4 Hz, 2H), 4.36 (t, *J* = 6.5 Hz, 4H), 3.94 (s, 6H), 3.94 (s, 6H), 2.66 – 2.37 (m, 12H), 2.03 – 1.91 (m, 4H).^13^C NMR (101 MHz, CDCl_3_) δ 166. 5, 153.0, 148.7, 123.6, 122.9, 112.0, 110.3, 63.3, 56.1, 56.1, 55.2, 53.2, 26.4. LC–MS (ESI^+^) *m/z* calcd. for C_28_H_38_N_2_O_8_ [(M + H)]^+^: 531.27; found: 531.25. HPLC *t*_R_: 6.736 and 6.928 min.

**piperazine-1,4-diylbis(propane-3,1-diyl) bis(3,5-dimethoxybenzoate) (6t).** Final compound **6t** was obtained from intermediate **2o** following general procedure G. Column chromatography with 0.5 to 4% MeOH in DCM as mobile phase. Transparent oil (501 mg, 0.94 mmol, 58%). ^1^H NMR (400 MHz, CDCl_3_) δ 7.17 (d, *J* = 2.4 Hz, 4H), 6.62 (t, *J* = 2.4 Hz, 2H), 4.36 (t, *J* = 6.5 Hz, 4H), 3.81 (s, 12H), 2.66 – 2.36 (m, 12H), 1.95 1.96 (p, *J* = 7.6, 6.5 Hz, 4H). ^13^C NMR (101 MHz, CDCl_3_) δ 166.0, 160.4, 132.0, 106.9, 105.2, 63.4, 55.3, 54.9, 53.0, 26.0. LC–MS (ESI +) m/z calcd. for C_28_H_38_N_2_O_8_ [(M + H)]^+^: 531.27; found: 531.25. HPLC *t*_R_: 7.833 min.

**3-(4-(3-((2-methoxybenzoyl)oxy)propyl)piperazin-1-yl)propyl 3,4-dimethoxybenzoate (6u).** Final compound **6j** was obtained from intermediates **2n** and **4a** following general procedure F. Column chromatography with 3 to 5% MeOH in DCM as mobile phase. Transparent oil (120 mg, 0.24 mmol, 43%). ^1^H NMR (400 MHz, CDCl_3_) δ 7.78 (dd, *J* = 7.9, 1.8 Hz, 1H), 7.68 (dd, *J* = 8.4, 2.0 Hz, 1H), 7.54 (d, *J* = 1.9 Hz, 1H), 7.47 (ddd, *J* = 8.5, 7.4, 1.8 Hz, 1H), 7.04 – 6.94 (m, 2H), 6.89 (d, *J* = 8.5 Hz, 1H), 4.41 – 4.31 (m, 4H), 3.94 (s, 3H), 3.93 (s, 3H), 3.90 (s, 3H), 2.61 – 2.40 (m, 12H), 2.01 – 1.90 (m, 4H). ^13^C NMR (101 MHz, CDCl_3_) δ 166.4, 166.3, 159.1, 152.9, 148.6, 133.5, 131.6, 123.5, 122.9, 120.3, 120.1, 112.0, 112.0, 110.2, 63.3, 63.3, 56.0, 56.0, 55.9, 55.2, 53.2, 26.4, 26.2. LC–MS (ESI +) *m/z* calcd. for C_27_H_36_N_2_O_7_ [(M + H)]^+^: 501.26; found: 501.20. HPLC *t*_R_: 2.491 min.

**3-(4-(3-((3,5-dimethoxybenzoyl)oxy)propyl)piperazin-1-yl)propyl 3,4-dimethoxybenzoate (6v).** Final compound **6j** was obtained from intermediates **2n** and **4b** following general procedure F. Automatic column chromatography with 0 to 5% MeOH in DCM as mobile phase on Biotage Isolera One. Transparent oil (676 mg, 1.27 mmol, 87%). ^1^H NMR (400 MHz, CDCl_3_) δ 7.66 (dd, *J* = 8.4, 2.0 Hz, 1H), 7.53 (d, *J* = 2.0 Hz, 1H), 7.16 (d, *J* = 2.4 Hz, 2H), 6.87 (d, *J* = 8.5 Hz, 1H), 6.62 (t, *J* = 2.4 Hz, 1H), 4.42 – 4.30 (m, 4H), 3.92 (s, 3H), 3.90 (s, 3H), 3.80 (s, 6H), 2.62 – 2.38 (m, 12H), 2.00 – 1.90 (m, 4H). ^13^C NMR (101 MHz, CDCl_3_) δ 165.8, 165.7, 160.2, 152.5, 148.2, 131.8, 123.0, 122.4, 111.5, 109.8, 106.7, 63.2, 62.8, 55.5, 55.5, 55.03, 54.7, 54.6, 52.8, 25.9, 25.8. LC–MS (ESI +) *m/z* calcd. for C_28_H_38_N_2_O_8_ [(M + H)]^+^: 531.27; found: 531.25. HPLC *t*_R_: 7.045 and 7.432 min.

**3-(fluorosulfonyl)benzoic acid (7a).** Intermediate **7a** was obtained from 3-(chlorosulfonyl)benzoic acid following general procedure J. White solid (602 mg, 2.94 mmol, 98%). ^1^H NMR (400 MHz, DMSO-d_6_) δ 13.87 (br s, 1H), 8.50 – 8.43 (m, 2H), 8.42 – 8.38 (m, 1H), 7.95 (t, *J* = 7.8 Hz, 1H). LC–MS (ESI-) *m/z* calcd. for C_7_H_5_FO_4_S [(M-H)]^−^: 202.98; found: 202.95. HPLC *t*_R_: 8.100 min.

**4-(fluorosulfonyl)benzoic acid (7b).** Intermediate **7b** was obtained from 4-(chlorosulfonyl)benzoic acid following general procedure J. White solid (961 mg, 4.71 mmol, 94%). ^1^H NMR (400 MHz, CDCl_3_) δ 8.38 – 8.32 (m, 2H), 8.18 – 8.12 (m, 2H). LC–MS (ESI-) *m/z* calcd. for C_7_H_5_FO_4_S [(M-H)]^−^: 202.98; found: 203.00. HPLC *t*_R_: 8.464 min.

**4-isothiocyanatobenzoic acid (7c).** A stirred solution of thiophosgene (0.15 mL, 2.00 mmol, 1.0 equiv) in acetone (2.00 mL) was allowed to cool down to 0 °C followed by addition of a solution of 4-aminobenzoic acid (274 mg, 2.00 mmol, 1.0 equiv) in acetone (2.00 mL). The mixture was allowed to stir at 0 °C for 3 h and at rt for 16 h. The reaction mixture was concentrated *in vacuo*, dissolved in EtOAc (20 mL) and washed with sat. NaHCO_3_ solution (20 mL). The aqueous phase was acidified to pH 3 by dropwise addition of 3 M aqueous HCl and extracted three times with EtOAc (20 mL). The combined organic phase was dried over MgSO_4_, filtrated and concentrated *in vacuo* to provide intermediate **7c** as a yellow solid. (136 mg, 0.76, 38%). ^1^H NMR (400 MHz, DMSO-d_6_) δ 7.98 (d, *J* = 8.4 Hz, 2H), 7.53 (d, *J* = 8.7 Hz, 2H). LC–MS (ESI-) *m/z* calcd. for C_8_H_5_NO_2_S [(M-H)]^−^: 178.00; found: 177.90. HPLC *t*_R_: 9.494 min.

**3-((tert-butoxycarbonyl)amino)benzoic acid (7d).** Intermediate **7d** was obtained from 3-aminobenzoic acid following general procedure K with Et_3_N. White solid (quant.). ^1^H NMR (400 MHz, CDCl_3_) δ 8.00 (t, *J* = 2.0 Hz, 1H), 7.79 – 7.70 (m, 2H), 7.41 (t, *J* = 7.9 Hz, 1H), 6.65 (br s, 1H), 1.54 (s, 9H). LC–MS (ESI-) *m/z* calcd. for C_12_H_15_NO_4_ [(M-H)]^−^: 236.09; found: 236.05. HPLC *t*_R_: 9.131 min.

**4-((tert-butoxycarbonyl)amino)benzoic acid (7e).** Intermediate **7e** was obtained from 4-aminobenzoic acid following general procedure K with Et_3_N. White solid (quant.). ^1^H NMR (400 MHz, CDCl_3_) δ 8.16 – 7.93 (m, 2H), 7.64 – 7.34 (m, 2H), 6.83 (br s, 1H), 1.54 (s, 9H). LC–MS (ESI-) *m/z* calcd. for C_12_H_15_NO_4_ [(M-H)]^−^: 236.09; found: 236.05. HPLC *t*_R_: 9.117 min.

**4-((tert-butoxycarbonyl)amino)-3-methoxybenzoic acid (7f).** Intermediate **7f** was obtained from 4-amino-3-methoxybenzoic acid following general procedure K with NaOH. Brown solid (1.86 g, 6.95 mmol, 70%). ^1^H NMR (400 MHz, CDCl_3_) δ 8.04 (d, *J* = 8.4 Hz, 1H), 7.97 (br s, 1H), 7.63 (dd, *J* = 8.4, 1.9 Hz, 1H), 7.57 (d, *J* = 1.8 Hz, 1H), 3.93 (s, 3H), 1.53 (s, 9H). LC–MS (ESI-) *m/z* calcd. for C_13_H_17_NO_5_ [(M-H)]^−^: 266.10; found: 266.00. HPLC *t*_R_: 9.926 min.

**4-(((tert-butoxycarbonyl)amino)methyl)benzoic acid (7g).** Intermediate **7g** was obtained from 4-(aminomethyl)benzoic acid following general procedure K with NaOH. White solid (1.77 g, 7.06 mmol, 88%). ^1^H NMR (400 MHz, MeOD) δ 7.98 (d, *J* = 8.0 Hz, 2H), 7.37 (d, *J* = 8.0 Hz, 2H), 4.29 (s, 2H), 1.46 (s, 9H). LC–MS (ESI-) *m/z* calcd. for C_13_H_17_NO_4_ [(M-H)]^−^: 250.11; found: 250.10. HPLC *t*_R_: 8.717 min.

**4-(2-((tert-butoxycarbonyl)amino)ethyl)benzoic acid (7h).** Intermediate **7h** was obtained from 4-(2-aminoethyl)benzoic acid hydrochloride following general procedure K with NaOH. White solid (945 mg, 3.56 mmol, 89%). ^1^H NMR (400 MHz, MeOD) δ 7.97 (d, *J* = 8.3 Hz, 2H), 7.34 (d, *J* = 8.2 Hz, 2H), 3.30 (t, *J* = 7.4 Hz, 2H), 2.85 (t, *J* = 7.3 Hz, 2H), 1.43 (s, 9H). LC–MS (ESI-) *m/z* calcd. for C_14_H_19_NO_4_ [(M-H)]^−^: 264.12; found: 264.10. HPLC *t*_R_: 9.064 min.

**2-bromoethyl 3-(fluorosulfonyl)benzoate (8a).** Intermediate **8a** was obtained from **7a** and 2-bromoethan-1-ol following general procedure C. Column chromatography with 30% EtOAc in PE as mobile phase. Transparent oil (259 mg, 0.83 mmol, 82%). ^1^H NMR (400 MHz, CDCl_3_) δ 8.69 (t, *J* = 1.8 Hz, 1H), 8.48 (dt, *J* = 7.9, 1.5 Hz, 1H), 8.23 (ddd, *J* = 7.9, 2.0, 1.2 Hz, 1H), 7.79 (t, *J* = 7.7 Hz, 1H), 4.71 (t, *J* = 6.0 Hz, 2H), 3.69 (t, *J* = 6.0 Hz, 2H).

**4-bromobutyl 3-(fluorosulfonyl)benzoate (8b).** Intermediate **8b** was obtained from **7a** and 4-bromobutan-1-ol following general procedure C. Column chromatography with 20% EtOAc in PE as mobile phase. Transparent oil (294 mg, 0.87 mmol, 83%). ^1^H NMR (400 MHz, CDCl_3_) δ 8.65 (t, *J* = 1.8 Hz, 1H), 8.46 (dt, *J* = 7.9, 1.4 Hz, 1H), 8.21 (ddd, *J* = 7.9, 2.0, 1.2 Hz, 1H), 7.79 (t, *J* = 8.0, 0.7 Hz, 1H), 4.45 (t, *J* = 6.1 Hz, 2H), 3.51 (t, *J* = 6.2 Hz, 2H), 2.14 – 1.94 (m, 4H).

**2-bromoethyl 4-(fluorosulfonyl)benzoate (8c).** Intermediate **8c** was obtained from **7b** and 2-bromoethan-1-ol following general procedure C. Column chromatography with 10% to 20% EtOAc in PE as mobile phase. Transparent oil (503 mg, 1.62 mmol, 50%). ^1^H NMR (400 MHz, CDCl_3_) δ 8.37 – 8.30 (m, 2H), 8.16 – 8.08 (m, 2H), 4.72 (t, *J* = 6.1 Hz, 2H), 3.71 (t, *J* = 6.0 Hz, 2H).

**3-bromopropyl 4-(fluorosulfonyl)benzoate (8d).** Intermediate **8d** was obtained from **7h** and 3-bromopropan-1-ol following general procedure C. Column chromatography with 20% EtOAc in PE as mobile phase. Transparent oil (756 mg, 2.33 mmol, 99%). ^1^H NMR (400 MHz, CDCl_3_) δ 8.33 – 8.25 (m, 2H), 8.11 (d, *J* = 8.5 Hz, 2H), 4.56 (t, *J* = 6.1 Hz, 2H), 3.57 (t, *J* = 6.4 Hz, 2H), 2.37 (p, *J* = 6.3 Hz, 2H).

**4-bromobutyl 4-(fluorosulfonyl)benzoate (8e).** Intermediate **8e** was obtained from **7b** and 4-bromobutan-1-ol following general procedure C. Column chromatography with 10% EtOAc in PE as mobile phase. Transparent oil (227 mg, 0.67 mmol, 41%). ^1^H NMR (400 MHz, CDCl_3_) δ 8.32 – 8.26 (m, 2H), 8.13 – 8.07 (m, 2H), 4.44 (t, *J* = 6.1 Hz, 2H), 3.50 (t, *J* = 6.3 Hz, 2H), 2.11 – 1.94 (m, 4H).

**3-bromopropyl 3-((tert-butoxycarbonyl)amino)benzoate (8f).** Intermediate **8f** was obtained from **7d** and 3-bromopropan-1-ol following general procedure L. Column chromatography with 5 to 15% EtOAc in PE as mobile phase. Yellow oil (157 mg, 0.44 mmol, 15%). ^1^H NMR (400 MHz, CDCl_3_) δ 7.94 – 7.89 (m, 1H), 7.78 – 7.70 (m, 1H), 7.70 (dt, *J* = 7.7, 1.3 Hz, 1H), 7.37 (t, *J* = 7.9 Hz, 1H), 6.79 (s, 1H), 4.46 (t, *J* = 6.0 Hz, 2H), 3.54 (t, *J* = 6.6 Hz, 2H), 2.37 – 2.19 (m, 2H), 1.53 (s, 9H).

**3-bromopropyl 4-((tert-butoxycarbonyl)amino)benzoate (8g).** Intermediate **8g** was obtained from **7e** and 3-bromopropan-1-ol following general procedure L. Column chromatography with 5 to 15% EtOAc in PE as mobile phase. Yellow oil (231 mg, 0.65 mmol, 22%). ^1^H NMR (400 MHz, CDCl_3_) δ 8.00 – 7.92 (m, 2H), 7.49 – 7.42 (m, 2H), 6.89 (s, 1H), 4.44 (t, *J* = 6.1 Hz, 2H), 3.54 (t, *J* = 6.6 Hz, 2H), 2.31 (p, *J* = 6.3 Hz, 2H), 1.52 (s, 9H).

**4-bromobutyl 4-((tert-butoxycarbonyl)amino)benzoate (8h).** Intermediate **8h** was obtained from **7e** and 4-bromobutan-1-ol following general procedure L. Column chromatography with 5 to 10% EtOAc in PE as mobile phase. White solid (332 mg, 0.89 mmol, 30%). ^1^H NMR (400 MHz, CDCl_3_) δ 8.01 – 7.92 (m, 2H), 7.54 – 7.45 (m, 2H), 7.28 (br s, 1H), 4.33 (t, *J* = 6.2 Hz, 2H), 3.47 (t, *J* = 6.5 Hz, 2H), 2.05 – 1.97 (m, 2H), 1.97 – 1.88 (m, 2H), 1.51 (s, 9H).

**3-bromopropyl 4-((tert-butoxycarbonyl)amino)-3-methoxybenzoate (8i).** Intermediate **8i** was obtained from **7f** and 3-bromopropan-1-ol following general procedure L. Column chromatography with 5 to 15% EtOAc in PE as mobile phase. Yellow oil (321 mg, 0.83 mmol, 28%). ^1^H NMR (400 MHz, CDCl_3_) δ 8.17 (d, *J* = 8.5 Hz, 1H), 7.65 (dd, *J* = 8.5, 1.8 Hz, 1H), 7.50 (d, *J* = 1.8 Hz, 1H), 7.30 (br s, 1H), 4.44 (t, *J* = 6.1 Hz, 2H), 3.93 (s, 3H), 3.54 (t, *J* = 6.6 Hz, 2H), 2.32 (p, *J* = 6.3 Hz, 2H), 1.53 (s, 9H).

**3-bromopropyl 4-(((tert-butoxycarbonyl)amino)methyl)benzoate (8j).** Intermediate **8j** was obtained from **7g** and 3-bromopropan-1-ol following general procedure L in the presence of pyridine (6.0 equiv). Column chromatography with 5 to 10% EtOAc in PE as mobile phase. White solid (743 mg, 2.00 mmol, 57%). ^1^H NMR (400 MHz, CDCl_3_) δ 7.98 (d, *J* = 8.3 Hz, 2H), 7.35 (d, *J* = 8.2 Hz, 2H), 5.16 (br s, 1H), 4.45 (t, *J* = 6.0 Hz, 2H), 4.34 (d, *J* = 6.6 Hz, 2H), 3.54 (t, *J* = 6.5 Hz, 2H), 2.31 (p, *J* = 6.3 Hz, 2H), 1.46 (s, 9H).

**3-bromopropyl 4-(2-((tert-butoxycarbonyl)amino)ethyl)benzoate (8k).** Intermediate **8k** was obtained from **7h** and 3-bromopropan-1-ol following general procedure L in the presence of pyridine (6.0 equiv). Column chromatography with 5 to 10% EtOAc in PE as mobile phase. White solid (192 mg, 0.50 mmol, 17%). ^1^H NMR (400 MHz, CDCl_3_) δ 7.97 (d, *J* = 8.4 Hz, 2H), 7.27 (d, *J* = 8.2 Hz, 2H), 4.72 (br t, *J* = 6.2 Hz, 1H), 4.45 (t, *J* = 6.0 Hz, 2H), 3.55 (t, *J* = 6.6 Hz, 2H), 3.39 (q, *J* = 6.8 Hz, 2H), 2.87 (t, *J* = 7.0 Hz, 2H), 2.32 (p, *J* = 6.3 Hz, 2H), 1.43 (s, 9H).

**3-(4-(2-((3-(fluorosulfonyl)benzoyl)oxy)ethyl)piperazin-1-yl)propyl 3,4,5-trimethoxybenzoate (9a).** Final compound **9a** was obtained from intermediates **8a** and **4c** following general procedure F. Column chromatography with 6 to 10% MeOH in EtOAc as mobile phase. Transparent oil (13.2 mg, 0.02 mmol, 4%). ^1^H NMR (400 MHz, CDCl_3_) δ 8.66 (t, *J* = 1.8 Hz, 1H), 8.43 (dt, *J* = 7.9, 1.4 Hz, 1H), 8.20 (ddd, *J* = 7.9, 2.0, 1.2 Hz, 1H), 7.76 (t, *J* = 8.4 Hz, 1H), 7.29 (s, 2H), 4.52 (t, *J* = 5.9 Hz, 2H), 4.37 (t, *J* = 6.6 Hz, 2H), 3.91 (s, 9H), 2.81 (t, *J* = 6.0 Hz, 2H), 2.72–2.40 (m, 10H), 2.00 (p, *J* = 6.8 Hz, 2H). ^13^C NMR (101 MHz, CDCl_3_) δ 166.4, 164.2, 153.0, 136.5, 132.3, 130.2, 129.7, 125.4, 106.9, 63.6, 63.4, 61.1, 56.6, 56.4, 55.2, 53.4, 53.2, 26.3. LC–MS (ESI +) *m*/*z* calcd. for C_26_H_33_FN_2_O_9_S [(M + H)]^+^: 569.20; found: 569.15. HPLC *t*_R_: 7.638 min.

**3-(4-(3-((3-(fluorosulfonyl)benzoyl)oxy)propyl)piperazin-1-yl)propyl 3,4,5-trimethoxybenzoate (9b).** Final compound **9b** was obtained from intermediate **5** and **7a** following general procedure I. Column chromatography with 3% MeOH in DCM as mobile phase. Transparent oil (30.3 mg, 0.05 mmol, 5%).^1^H NMR (400 MHz, CDCl_3_) δ 8.65 (t, *J* = 1.6 Hz, 1H), 8.43 (dt, *J* = 8.0, 1.6 Hz, 1H), 8.20 (ddd, *J* = 8.0, 2.0, 1.2 Hz, 1H), 7.75 (t, *J* = 7.8 Hz, 1H), 7.29 (s, 2H), 4.45 (t, *J* = 6.6 Hz, 2H), 4.37 (t, *J* = 6.6 Hz, 2H), 3.91 (s, 6H), 3.91 (s, 3H), 2.85 – 2.24 (m, 12H), 2.09 – 1.87 (m, 4H). ^13^C NMR (101 MHz, CDCl_3_) δ 166.3, 164.2, 153.0, 136.4, 132.4, 132.3, 130.2, 129.6, 125.5, 106.9, 64.7, 63.7, 61.1, 56.4, 55.2, 55.0, 53.3, 26.4, 26.2. LC–MS (ESI +) *m*/*z* calcd. for C_27_H_35_FN_2_O_9_S [(M + H)]^+^: 583.21; found: 583.25. HPLC *t*_R_: 7.585 min.

**3-(4-(4-((3-(fluorosulfonyl)benzoyl)oxy)butyl)piperazin-1-yl)propyl 3,4,5-trimethoxybenzoate (9c).** Final compound **9c** was obtained from intermediates **8b** and **4c** following general procedure F. Column chromatography with 4 to 8% MeOH in DCM as mobile phase. Transparent oil (40.1 mg, 0.06 mmol, 13%). ^1^H NMR (400 MHz, CDCl_3_) δ 8.65 (t, *J* = 1.8 Hz, 1H), 8.44 (dt, *J* = 7.9, 1.5 Hz, 1H), 8.20 (ddd, *J* = 7.9, 2.0, 1.2 Hz, 1H), 7.75 (t, *J* = 7.9 Hz, 1H), 7.29 (s, 2H), 4.41 (t, *J* = 6.6 Hz, 2H), 4.37 (t, *J* = 6.6 Hz, 2H), 3.91 (s, 9H), 2.63 – 2.33 (m, 12H), 2.01 – 1.93 (m, 2H), 1.84 (p, *J* = 8.0, 7.0, 6.5 Hz, 2H), 1.70 – 1.60 (m, 2H). ^13^C NMR (101 MHz, CDCl_3_) δ 166.3, 164.2, 153.0, 136.4, 133.9, 132.4, 132.2, 130.1, 129.6, 125.5, 106.9, 66.1, 63.7, 61.0, 58.1, 56.4, 55.2, 53.3, 26.8, 26.4, 23.5. LC–MS (ESI +) *m*/*z* calcd. for C_28_H_37_FN_2_O_9_S [(M + H)]^+^: 597.23; found: 597.35. HPLC *t*_R_: 7.675 min.

**3-(4-(2-((4-(fluorosulfonyl)benzoyl)oxy)ethyl)piperazin-1-yl)propyl 3,4,5-trimethoxybenzoate (9d).** Final compound **9d** was obtained from intermediates **8c** and **4c** following general procedure F. Column chromatography with 4 to 8% MeOH in DCM as mobile phase. Transparent oil (21.6 mg, 0.03 mmol, 4%). ^1^H NMR (400 MHz, CDCl_3_) δ 8.32 – 8.24 (m, 2H), 8.13 – 8.07 (m, 2H), 7.29 (s, 2H), 4.52 (t, *J* = 5.9 Hz, 2H), 4.37 (t, *J* = 6.6 Hz, 2H), 3.91 (s, 9H), 2.81 (t, *J* = 5.9 Hz, 2H), 2.76 – 2.29 (m, 10H), 1.97 (p, *J* = 6.8 Hz, 2H). ^13^C NMR (101 MHz, CDCl_3_) δ 166.4, 164.5, 153.0, 136.7, 130.8, 128.7, 125.5, 106.9, 63.7, 63.6, 61.1, 56.6, 56.4, 55.2, 53.5, 53.3, 26.4. LC–MS (ESI +) *m*/*z* calcd. for C_26_H_33_FN_2_O_9_S [(M + H)]^+^: 569.19; found: 569.60. HPLC *t*_R_: 7.677 min.

**3-(4-(3-((4-(fluorosulfonyl)benzoyl)oxy)propyl)piperazin-1-yl)propyl 3,4,5-trimethoxybenzoate (9e).** Final compound **9e** was obtained from intermediates **8d** and **4c** following general procedure F. Column chromatography with 2 to 3% MeOH in DCM as mobile phase. Transparent oil (12.8 mg, 0.02 mmol, 1%). ^1^H NMR (400 MHz, CDCl_3_) δ 8.27 (d, *J* = 7.9 Hz, 2H), 8.10 (d, *J* = 8.7 Hz, 2H), 7.29 (s, 2H), 4.45 (t, *J* = 6.5 Hz, 2H), 4.37 (t, *J* = 6.6 Hz, 2H), 3.91 (s, 9H), 2.87 – 2.22 (m, 12H), 2.08 – 1.91 (m, 4H). ^13^C NMR (101 MHz, CDCl_3_) δ 166.4, 164.6, 153.1, 136.8, 130.8, 128.7, 125.4, 106.9, 64.6, 63.6, 61.1, 56.4, 55.2, 55.0, 53.2, 26.3, 26.2. LC–MS (ESI +) *m*/*z* calcd. for C_27_H_35_FN_2_O_9_S [(M + H)]^+^: 583.21; found: 583.20. HPLC *t*_R_: 7.636 min.

**3-(4-(4-((4-(fluorosulfonyl)benzoyl)oxy)butyl)piperazin-1-yl)propyl 3,4,5-trimethoxybenzoate (9f).** Final compound **9f** was obtained from intermediates **8e** and **4c** following general procedure F. Column chromatography with 4 to 8% MeOH in DCM as mobile phase. Transparent oil (23.3 mg, 0.04 mmol, 10%). ^1^H NMR (400 MHz, CDCl_3_) δ [ppm] 8.31 – 8.25 (m, 2H), 8.13 – 8.06 (m, 2H), 7.29 (s, 2H), 4.41 (t, *J* = 6.5 Hz, 2H), 4.37 (t, *J* = 6.6 Hz, 2H), 3.91 (s, 9H), 2.84 – 2.27 (m, 12H), 1.98 (p, *J* = 6.8 Hz, 2H), 1.83 (p, *J* = 7.1 Hz, 2H), 1.72 – 1.58 (m, 2H). ^13^C NMR (101 MHz, CDCl_3_) δ 166.4, 164.6, 153.0, 136.9, 130.8, 128.6, 125.5, 106.9, 66.1, 63.7, 61.1, 58.1, 56.4, 55.2, 53.3, 26.8, 26.4, 23.5. LC–MS (ESI +) *m*/*z* calcd. for C_28_H_37_FN_2_O_9_S [(M + H)]^+^: 597.23; found: 597.25. HPLC *t*_R_: 7.762 min.

**3-(4-(3-((4-isothiocyanatobenzoyl)oxy)propyl)piperazin-1-yl)propyl 3,4,5-trimethoxybenzoate (9g).** To a stirred solution of benzoic acid **7c** (136 mg, 0.76 mmol, 1.0 equiv) in DCM (7.5 mL) was added EDC·HCl (291 mg, 1.62 mmol, 2.0 equiv) and DMAP (cat.). Subsequently intermediate **5** (301 mg, 0.76 mmol, 1.0 equiv) was added and the reaction mixture was stirred for 16 h at rt under N_2_ atmosphere. The mixture was diluted with DCM (20 mL) after which the organic phase was washed twice with H_2_O (20 mL). The combined aqueous phases were extracted with DCM (20 mL) and the combined organic phases were dried over MgSO_4_, filtrated and concentrated *in vacuo*. Flash column chromatography on silica gel using a gradient of 0 to 5% MeOH in DCM as mobile phase provided final compound **9g**. Yellow oil (34.2 mg, 0.06 mmol, 8%). ^1^H NMR (400 MHz, CDCl_3_) δ 8.02 (d, *J* = 8.7 Hz, 2H), 7.29 (s, 2H), 7.31 – 7.23 (m, 2H), 4.37 (t, *J* = 6.6 Hz, 4H), 3.91 (s, 6H), 3.91 (s, 3H), 2.68 – 2.41 (m, 12H), 2.04 – 1.91 (m, 4H). ^13^C NMR (101 MHz, CDCl_3_) δ 166.3, 165.5, 153.0, 142.3, 137.9, 135.7, 131.1, 128.9, 125.8, 125.4, 106.9, 63.9, 63.7, 61.0, 56.4, 55.2, 55.1, 53.2, 26.3, 26.3. LC–MS (ESI +) *m*/*z* calcd. for C_28_H_35_N_3_O_7_S [(M + H)]^+^: 558.22; found: 558.15. HPLC *t*_R_: 8.163 min.

**3-(4-(3-((3-((tert-butoxycarbonyl)amino)benzoyl)oxy)propyl)piperazin-1-yl)propyl 3,4,5-trimethoxybenzoate (9h).** Intermediate **9h** was obtained from intermediates **8f** and **4c** following general procedure F. Column chromatography with 0 to 4% MeOH in DCM as mobile phase. Yellow oil (134 mg, 0.22 mmol, 59%). ^1^H NMR (400 MHz, CDCl_3_) δ 7.91 (t, *J* = 1.9 Hz, 1H), 7.76 – 7.65 (m, 2H), 7.36 (t, *J* = 8.0 Hz, 1H), 7.29 (s, 2H), 6.77 (s, 1H), 4.37 (td, *J* = 6.5, 4.1 Hz, 4H), 3.91 (s, 9H), 2.72 – 2.37 (m, 12H), 2.02 – 1.90 (m, 4H), 1.52 (s, 9H). LC–MS (ESI +) *m/z* calcd. for C_32_H_45_N_3_O_9_ [(M + H)]^+^: 616.32; found: 616.30. HPLC *t*_R_: 7.955 min.

**3-(4-(3-((4-((tert-butoxycarbonyl)amino)benzoyl)oxy)propyl)piperazin-1-yl)propyl 3,4,5-trimethoxybenzoate (9i).** Intermediate **9i** was obtained from intermediates **8g** and **4c** following general procedure F. Column chromatography with 0 to 4% MeOH in DCM as mobile phase. Yellow oil (222 mg, 0.36 mmol, 84%). ^1^H NMR (400 MHz, CDCl_3_) δ 8.00 – 7.92 (m, 2H), 7.46 (d, *J* = 8.7 Hz, 2H), 7.29 (s, 2H), 7.06 (s, 1H), 4.36 (dt, *J* = 11.3, 6.5 Hz, 4H), 3.91 (s, 9H), 2.77 – 2.31 (m, 12H), 2.06 – 1.91 (m, 4H), 1.52 (s, 9H). LC–MS (ESI +) *m/z* calcd. for C_32_H_45_N_3_O_9_ [(M + H)]^+^: 616.32; found: 616.35. HPLC *t*_R_: 7.955 min.

**3-(4-(4-((4-((tert-butoxycarbonyl)amino)benzoyl)oxy)butyl)piperazin-1-yl)propyl 3,4,5-trimethoxybenzoate (9j).** Intermediate **9j** was obtained from intermediates **8h** and **4c** following general procedure F. Column chromatography with 0 to 4% MeOH in DCM as mobile phase. Yellow oil (226 mg, 0.36 mmol, 60%). ^1^H NMR (400 MHz, CDCl_3_) δ 7.96 (d, *J* = 8.7 Hz, 2H), 7.47 (d, *J* = 8.7 Hz, 2H), 7.29 (s, 2H), 7.20 (br s, 1H), 4.38 (t, *J* = 6.5 Hz, 2H), 4.31 (t, *J* = 6.4 Hz, 2H), 3.91 (s, 3H), 3.90 (s, 6H), 2.86 – 2.22 (m, 12H), 2.03 – 1.94 (m, 2H), 1.84 – 1.73 (m, 2H), 1.71 – 1.61 (m, 2H), 1.51 (s, 9H). LC–MS (ESI +) *m*/*z* calcd. for C_33_H_47_N_3_O_9_ [(M + H)]^+^: 630.34; found: 630.30. HPLC *t*_R_: 8.078 min.

**3-(4-(3-((4-((tert-butoxycarbonyl)amino)-3-methoxybenzoyl)oxy)propyl)piperazin-1-yl)propyl 3,4,5-trimethoxybenzoate (9k).** Intermediate **9k** was obtained from intermediates **8i** and **4c** following general procedure F. Column chromatography with 0 to 4% MeOH in DCM as mobile phase. Yellow oil (255 mg, 0.40 mmol, 72%). ^1^H NMR (400 MHz, CDCl_3_) δ 8.16 (d, *J* = 8.5 Hz, 1H), 7.66 (dd, *J* = 8.5, 1.8 Hz, 1H), 7.51 (d, *J* = 1.7 Hz, 1H), 7.29 (s, 3H), 4.36 (q, *J* = 6.8 Hz, 4H), 3.93 (s, 3H), 3.91 (s, 6H), 3.91 (s, 3H), 2.70 – 2.43 (m, 12H), 2.05 – 1.92 (m, 4H), 1.53 (s, 9H). LC–MS (ESI +) *m*/*z* calcd. for C_33_H_47_N_3_O_10_ [(M + H)]^+^: 646.33; found: 646.35. HPLC *t*_R_: 8.326 min.

**3-(4-(3-((4-(((tert-butoxycarbonyl)amino)methyl)benzoyl)oxy)propyl)piperazin-1-yl)propyl 3,4,5-trimethoxybenzoate (9l).** Intermediate **9l** was obtained from intermediates **8j** and **4c** following general procedure F. Column chromatography with 1 to 3% MeOH in DCM as mobile phase. Yellow oil (254 mg, 0.40 mmol, 40%). ^1^H NMR (400 MHz, CDCl_3_) δ 7.98 (d, *J* = 8.1 Hz, 2H), 7.35 (d, *J* = 8.3 Hz, 2H), 7.29 (s, 2H), 5.41 (t, *J* = 6.1 Hz, 1H), 4.43 – 4.29 (m, 6H), 3.91 (s, 6H), 3.90 (s, 3H), 2.72 – 2.38 (m, 12H), 2.03 – 1.92 (m, 4H), 1.46 (s, 9H). LC–MS (ESI +) *m*/*z* calcd. for C_33_H_47_N_3_O_9_ [(M + H)]^+^: 630.34; found: 630.30. HPLC *t*_R_: 7.890 min.

**3-(4-(3-((4-(2-((tert-butoxycarbonyl)amino)ethyl)benzoyl)oxy)propyl)piperazin-1-yl)propyl 3,4,5-trimethoxybenzoate (9m).** Intermediate **9m** was obtained from intermediates **8k** and **4c** following general procedure F. Column chromatography with 0 to 4% MeOH in DCM as mobile phase. Yellow oil (132 mg, 0.21 mmol, 62%). ^1^H NMR (400 MHz, CDCl_3_) δ 7.97 (d, *J* = 8.2 Hz, 2H), 7.29 (s, 2H), 7.27 (d, *J* = 8.0 Hz, 2H), 4.69 (t, *J* = 6.0 Hz, 1H), 4.42 – 4.34 (m, 4H), 3.91 (s, 6H), 3.91 (s, 3H), 3.39 (q, *J* = 6.8 Hz, 2H), 2.86 (t, *J* = 7.1 Hz, 2H), 2.69 – 2.42 (m, 12H), 2.04 – 1.91 (m, 4H), 1.43 (s, 9H). LC–MS (ESI +) *m*/*z* calcd. for C_34_H_49_N_3_O_9_ [(M + H)]^+^: 644.35; found: 644.35. HPLC *t*_R_: 8.014 min.

**3-(4-(3-((3-acrylamidobenzoyl)oxy)propyl)piperazin-1-yl)propyl 3,4,5-trimethoxybenzoate (10a).** Final compound **10a** was obtained from intermediate **9h** following general procedure N. Column chromatography with 3 to 5% MeOH in DCM as mobile phase. Transparent oil (10.3 mg, 0.02 mmol, 8%). ^1^H NMR (400 MHz, CDCl_3_) δ 8.29 – 7.98 (m, 3H), 7.76 (dt, *J* = 7.8, 1.4 Hz, 1H), 7.41 (t, J = 8.2 Hz, 1H), 7.29 (s, 2H), 6.47 (dd, *J* = 16.9, 1.7 Hz, 1H), 6.37 (dd, *J* = 16.8, 9.9 Hz, 1H), 5.78 (dd, *J* = 9.8, 1.7 Hz, 1H), 4.41 – 4.30 (m, 4H), 3.91 (s, 9H), 2.95 – 2.48 (m, 12H), 2.10 – 1.96 (m, 4H). ^13^C NMR (101 MHz, CDCl_3_) δ 166.4, 166.2, 163.9, 153.0, 142.2, 138.4, 131.1, 131.0, 129.3, 128.3, 125.4, 124.6, 120.9 106.9, 63.7, 63.5, 61.0, 56.3, 55.1, 55.0, 53.0, 52.9, 26.2, 26.1. LC–MS (ESI +) *m*/*z* calcd. for C_30_H_39_N_3_O_8_ [(M + H)]^+^: 570.28; found: 570.25. HPLC *t*_R_: 7.006 min.

**3-(4-(3-((4-acrylamidobenzoyl)oxy)propyl)piperazin-1-yl)propyl 3,4,5-trimethoxybenzoate (10b).** Final compound **10b** was obtained from intermediate **9i** following general procedure N. Column chromatography with 1 to 6% MeOH in DCM as mobile phase. Transparent oil (80.7 mg, 0.14 mmol, 55%). ^1^H NMR (400 MHz, CDCl_3_) δ 8.17 (s, 1H), 7.99 (d, *J* = 8.7 Hz, 2H), 7.72 (d, *J* = 8.9 Hz, 2H), 7.29 (s, 2H), 6.47 (dd, *J* = 16.8, 1.4 Hz, 1H), 6.33 (dd, *J* = 16.9, 10.1 Hz, 1H), 5.79 (dd, *J* = 10.1, 1.4 Hz, 1H), 4.40 – 4.32 (m, 4H), 3.91 (s, 3H), 3.90 (s, 6H), 2.78 – 2.44 (m, 12H), 2.04 – 1.94 (m, 4H). ^13^C NMR (101 MHz, CDCl_3_) δ 166.4, 166.2, 163.9, 153.0, 142.4, 131.0, 130.9, 128.7, 125.8, 125.3, 119.2, 106.8, 63.5, 63.2, 61.0, 56.3, 55.0, 52.7, 26.0. LC–MS (ESI +) *m*/*z* calcd. for C_30_H_39_N_3_O_8_ [(M + H)]^+^: 570.28; found: 570.30. HPLC *t*_R_: 7.039 min.

**3-(4-(4-((4-acrylamidobenzoyl)oxy)butyl)piperazin-1-yl)propyl 3,4,5-trimethoxybenzoate (10c).** Final compound **10c** was obtained from intermediate **9j** following general procedure N. Column chromatography with 3 to 5% MeOH in DCM as mobile phase. Transparent oil (164 mg, 0.28 mmol, 78%). ^1^H NMR (400 MHz, CDCl_3_) δ 8.74 (s, 1H), 7.97 (d, *J* = 8.8 Hz, 2H), 7.76 (d, *J* = 8.8 Hz, 2H), 7.29 (s, 2H), 6.52 – 6.36 (m, 2H), 5.76 (dd, *J* = 8.9, 2.6 Hz, 1H), 4.37 (t, *J* = 6.5 Hz, 2H), 4.30 (t, *J* = 6.1 Hz, 2H), 3.91 (s, 3H), 3.90 (s, 6H), 2.87 – 2.43 (m, 12H), 2.00 (p, *J* = 6.7 Hz, 2H), 1.84 – 1.66 (m, 4H). ^13^C NMR (101 MHz, CDCl_3_) δ 166.3, 166.2, 164.1, 152.9, 142.6, 142.2, 131.1, 130.7, 128.5, 125.6, 125.3, 119.2, 106.8, 64.5, 63.4, 61.0, 57.8, 56.3, 54.8, 52.7, 52.3, 26.6, 26.0, 22.8. LC–MS (ESI +) *m*/*z* calcd. for C_31_H_41_N_3_O_8_ [(M + H)]^+^: 584.30; found: 584.25. HPLC *t*_R_: 7.149 min.

**3-(4-(3-((4-acrylamido-3-methoxybenzoyl)oxy)propyl)piperazin-1-yl)propyl 3,4,5-trimethoxybenzoate (10d).** Final compound **10d** was obtained from intermediate **9k** following general procedure N. Column chromatography with 3% MeOH in DCM as mobile phase. Transparent oil (83.5 mg, 0.14 mmol, 48%). ^1^H NMR (400 MHz, CDCl_3_) δ 8.56 (d, *J* = 8.5 Hz, 1H), 8.07 (s, 1H), 7.69 (dd, *J* = 8.5, 1.8 Hz, 1H), 7.55 (d, *J* = 1.8 Hz, 1H), 7.29 (s, 2H), 6.46 (dd, *J* = 16.8, 1.3 Hz, 1H), 6.32 (dd, *J* = 16.9, 10.1 Hz, 1H), 5.81 (dd, *J* = 10.1, 1.3 Hz, 1H), 4.41 – 4.33 (m, 4H), 3.96 (s, 3H), 3.91 (s, 6H), 3.91 (s, 3H), 2.93 – 2.38 (m, 12H), 2.03 – 1.93 (m, 4H). ^13^C NMR (126 MHz, CDCl_3_) δ 166.3, 166.3, 163.5, 153.0, 147.5, 142.2, 131.9, 131.3, 128.4, 125.4, 125.4, 123.4, 118.9, 110.7, 106.8, 63.6, 63.5, 61.0, 56.3, 56.1, 55.1, 53.1, 26.3. LC–MS (ESI +) *m*/*z* calcd. for C_31_H_41_N_3_O_9_ [(M + H)]^+^: 600.29; found: 600.30. HPLC *t*_R_: 6.878 and 7.263 min.

**3-(4-(3-((4-(acrylamidomethyl)benzoyl)oxy)propyl)piperazin-1-yl)propyl 3,4,5-trimethoxybenzoate (10e).** Final compound **10e** was obtained from intermediate **9l** following general procedure N. Column chromatography with 3 to 5% MeOH in DCM as mobile phase. Transparent oil (40.3 mg, 0.07 mmol, 17%). ^1^H NMR (400 MHz, CDCl_3_) δ 7.97 (d, *J* = 8.3 Hz, 2H), 7.35 (d, *J* = 8.4 Hz, 2H), 7.28 (s, 2H), 6.34 (dd, *J* = 17.0, 1.5 Hz, 1H), 6.30 (t, *J* = 6.2 Hz, 1H), 6.17 (dd, *J* = 17.0, 10.2 Hz, 1H), 5.69 (dd, *J* = 10.2, 1.5 Hz, 1H), 4.57 (d, *J* = 6.0 Hz, 2H), 4.39–4.33 (m, 4H), 3.91 (s, 6H), 3.90 (s, 3H), 2.79 – 2.51 (m, 12H), 2.05–1.95 (m, 4H). ^13^C NMR (101 MHz, CDCl_3_) δ 166.4, 166.3, 165.7, 153.0, 143.6, 142.3, 130.5, 130.0, 129.5, 127.7, 127.3, 125.3, 106.9, 63.4, 63.2, 61.0, 56.4, 55.0, 54.9, 52.7, 43.3, 26.0. LC–MS (ESI +) *m*/*z* calcd. for C_31_H_41_N_3_O_8_ [(M + H)]^+^: 584.30; found: 584.25. HPLC *t*_R_: 6.806 min.

**3-(4-(3-((4-(2-acrylamidoethyl)benzoyl)oxy)propyl)piperazin-1-yl)propyl 3,4,5-trimethoxybenzoate (10f).** Final compound **10f** was obtained from intermediate **9m** following general procedure N. Column chromatography with 3 to 6% MeOH in DCM as mobile phase. Transparent oil (34.8 mg, 0.06 mmol, 28%). ^1^H NMR (400 MHz, CDCl_3_) δ 7.98 (d, *J* = 8.3 Hz, 2H), 7.30 – 7.26 (m, 4H), 6.27 (dd, *J* = 17.0, 1.4 Hz, 1H), 6.04 (dd, *J* = 17.0, 10.3 Hz, 1H), 5.67 (t, *J* = 5.9 Hz, 1H), 5.64 (dd, *J* = 10.3, 1.4 Hz, 1H), 4.39 – 4.34 (m, 4H), 3.91 (s, 6H), 3.91 (s, 3H), 3.62 (q, *J* = 6.8 Hz, 2H), 2.93 (t, *J* = 7.0 Hz, 2H), 2.64 – 2.48 (m, 12H), 2.03 – 1.93 (m, 4H). ^13^C NMR (101 MHz, CDCl_3_) δ 166.5, 166.3, 165.7, 153.0, 144.4, 142.3, 130.7, 130.0, 128.9, 128.8, 126.8, 125.4, 106.9, 63.6, 63.4, 61.1, 56.4, 55.2, 53.1, 40.5, 35.8, 26.3. LC–MS (ESI +) *m*/*z* calcd. for C_32_H_43_N_3_O_8_ [(M + H)]^+^: 598.31; found: 598.30. HPLC *t*_R_: 6.968 min.

**4-(2-((tert-butoxycarbonyl)amino)ethoxy)-3,5-dimethoxybenzoic acid (11a).** Intermediate **11a** was obtained from tert-butyl (2-bromoethyl)carbamate following general procedure M. Column chromatography with 1 to 4% MeOH in DCM as mobile phase. Transparent oil (785 mg, 2.30 mmol, 77%). ^1^H NMR (400 MHz, CDCl_3_) δ 10.40 (s, 1H), 7.37 (s, 2H), 5.97 (t, *J* = 5.5 Hz, 1H), 4.15 (t, *J* = 4.9 Hz, 2H), 3.93 (s, 6H), 3.44 (q, *J* = 5.1 Hz, 2H), 1.47 (s, 9H). LC–MS (ESI-) *m/z* calcd. for C_16_H_23_NO_7_ [(M-H)]^−^: 340.14; found: 340.10. HPLC *t*_R_: 9.356 min.

**4-(3-((tert-butoxycarbonyl)amino)propoxy)-3,5-dimethoxybenzoic acid (11b).** Intermediate **11b** was obtained from tert-butyl (3-bromopropyl)carbamate following general procedure M. Column chromatography with 1 to 4% MeOH in DCM as mobile phase. Transparent oil (1.25 g, 3.52 mmol, 88%). ^1^H NMR (400 MHz, CDCl_3_) δ 9.91 (s, 1H), 7.37 (s, 2H), 5.95 (br t, *J* = 6.0 Hz, 1H), 4.14 (t, *J* = 5.8 Hz, 2H), 3.92 (s, 6H), 3.49 – 3.36 (m, 2H), 2.04 – 1.89 (m, 2H), 1.48 (s, 9H). LC–MS (ESI-) *m/z* calcd. for C_17_H_25_NO_7_ [(M-H)]^−^: 354.16; found: 354.10. HPLC *t*_R_: 9.600 min.

**3-bromopropyl 4-(2-((tert-butoxycarbonyl)amino)ethoxy)-3,5-dimethoxybenzoate (12a)**. Intermediate **12a** was obtained from intermediate **9a** following general procedure D. Column chromatography with 10 to 15% EtOAc in PE as mobile phase. Transparent oil (286 mg, 0.62 mmol, 45%). ^1^H NMR (400 MHz, CDCl_3_) δ 7.30 (s, 2H), 5.84 (t, *J* = 5.6 Hz, 1H), 4.51 – 4.44 (m, 2H), 4.12 (t, *J* = 4.9 Hz, 2H), 3.92 (s, 6H), 3.54 (t, *J* = 6.5 Hz, 2H), 3.40 (q, *J* = 5.2 Hz, 2H), 2.34 (p, *J* = 6.3 Hz, 2H), 1.45 (s, 9H). LC–MS (ESI +) *m*/*z* calcd. for C_19_H_28_BrNO_7_ [(M + H)]^+^: 462.11; found: 462.10. HPLC *t*_R_: 11.734 min.

**3-bromopropyl 4-(3-((tert-butoxycarbonyl)amino)propoxy)-3,5-dimethoxybenzoate (12b).** Intermediate **12b** was obtained from intermediate **9b** following general procedure D. Column chromatography with 0.5 to 1.5% MeOH in DCM as mobile phase. Transparent oil (800 mg, 1.68 mmol, 39%). ^1^H NMR (400 MHz, CDCl_3_) δ 7.30 (s, 2H), 5.73 (br s, 1H), 4.47 (t, *J* = 6.1 Hz, 2H), 4.10 (t, *J* = 5.7 Hz, 2H), 3.92 (s, 6H), 3.55 (td, *J* = 6.5, 4.6 Hz, 4H), 2.34 (p, *J* = 6.3 Hz, 2H), 2.10 (p, *J* = 6.2 Hz, 2H), 1.46 (s, 9H). LC–MS (ESI +) *m*/*z* calcd. for C_20_H_30_BrNO_7_ [(M + H)]^+^: 476.13; found: 476.10. HPLC *t*_R_: 11.876 min.

**3-(4-(3-((4-(2-((tert-butoxycarbonyl)amino)ethoxy)-3,5-dimethoxybenzoyl)oxy)propyl)piperazin-1-yl)propyl 3,4,5-trimethoxybenzoate (13a).** Intermediate **13a** was obtained from intermediates **12a** and **4c** following general procedure F. Automatic column chromatography with 3 to 5% MeOH in DCM as mobile phase on Biotage Isolera One. Yellow oil (136 mg, 0.19 mmol, 37%). ^1^H NMR (400 MHz, CDCl_3_) δ 7.29 (s, 4H), 5.86 (t, *J* = 5.5 Hz, 1H), 4.38 (t, *J* = 6.6, 1.4 Hz, 4H), 4.12 (t, *J* = 4.9 Hz, 2H), 3.92 (s, 6H), 3.91 (s, 6H), 3.91 (s, 3H), 3.40 (q, *J* = 5.1 Hz, 2H), 2.75 – 2.36 (m, 12), 2.04 – 1.92 (m, 4H), 1.46 (s, 9H). LC–MS (ESI +) *m*/*z* calcd. for C_36_H_53_N_3_O_12_ [(M + H)]^+^: 720.37; found: 720.30. HPLC *t*_R_: 8.033 min.

**3-(4-(3-((4-(3-((tert-butoxycarbonyl)amino)propoxy)-3,5-dimethoxybenzoyl)oxy)propyl)piperazin-1-yl)propyl 3,4,5-trimethoxybenzoate (13b).** Intermediate **13b** was obtained from intermediates **12b** and **4c** following general procedure F. Automatic column chromatography with 3 to 5% MeOH in DCM as mobile phase on Biotage Isolera One. Yellow oil (305 mg, 0.42 mmol, 53%). ^1^H NMR (400 MHz, CDCl_3_) δ 7.30 (s, 2H), 7.30 (s, 2H), 5.79 (t, *J* = 5.8 Hz, 1H), 4.38 (t, *J* = 6.5 Hz, 4H), 4.11 (t, *J* = 5.7 Hz, 2H), 3.92 (s, 6H), 3.91 (s, 6H), 3.91 (s, 3H), 3.42 (q, *J* = 6.1 Hz, 2H), 2.81 – 2.31 (m, 12H), 2.07 – 1.87 (m, 6H), 1.47 (s, 9H). LC–MS (ESI +) *m*/*z* calcd. for C_37_H_55_N_3_O_12_ [(M + H)]^+^: 734.39; found: 734.40. HPLC *t*_R_: 8.259 min.

**3-(4-(3-((4-(2-acrylamidoethoxy)-3,5-dimethoxybenzoyl)oxy)propyl)piperazin-1-yl)propyl 3,4,5-trimethoxybenzoate (14a).** Final compound **14a** was obtained from intermediate **13a** following general procedure N. Column chromatography with 4 to 8% MeOH in DCM as mobile phase. Transparent oil (65.6 mg, 0.09 mmol, 15%). ^1^H NMR (400 MHz, CDCl_3_) δ 7.31 (s, 2H), 7.29 (s, 2H), 6.87 (br t, *J* = 5.4 Hz, 1H), 6.30 (dd, *J* = 17.0, 1.5 Hz, 1H), 6.15 (dd, *J* = 17.0, 10.2 Hz, 1H), 5.67 (dd, *J* = 10.2, 1.6 Hz, 1H), 4.41 – 4.35 (m, 4H), 4.17 (t, *J* = 5.0 Hz, 2H), 3.92 (s, 6H), 3.91 (s, 9H), 3.63 (q, *J* = 5.2 Hz, 2H), 2.70 – 2.41 (m, 12H), 1.99 (p, *J* = 6.8 Hz, 4H). ^13^C NMR (101 MHz, CDCl_3_) δ 166.3, 166.1, 165.5, 153.0, 152.9, 142.2, 140.5, 131.2, 126.2, 126.0, 125.4, 106.8, 72.7, 63.8, 63.6, 61.0, 56.4, 56.3, 55.1, 53.2, 39.4, 26.3. LC–MS (ESI +) *m*/*z* calcd. for C_34_H_47_N_3_O_11_ [(M + H)]^+^: 674.33; found: 674.35. HPLC *t*_R_: 7.183 min.

**3-(4-(3-((4-(3-acrylamidopropoxy)-3,5-dimethoxybenzoyl)oxy)propyl)piperazin-1-yl)propyl 3,4,5-trimethoxybenzoate (14b).** Final compound **14b** was obtained from intermediate **13b** following general procedure N. Column chromatography with 3 to 6% MeOH in DCM as mobile phase. Transparent oil (105 mg, 0.15 mmol, 25%). ^1^H NMR (400 MHz, DMSO-d_6_) δ 8.14 (t, *J* = 5.6 Hz, 1H), 7.26 (s, 2H), 7.26 (s, 2H), 6.21 (dd, *J* = 17.1, 10.1 Hz, 1H), 6.06 (dd, *J* = 17.1, 2.3 Hz, 1H), 5.56 (dd, *J* = 10.1, 2.3 Hz, 1H), 4.34 (t, *J* = 6.1 Hz, 4H), 3.97 (t, *J* = 6.3 Hz, 2H), 3.85 (s, 6H), 3.84 (s, 6H), 3.73 (s, 3H), 3.33 – 3.23 (m, 14H), 2.27 – 2.12 (m, 4H), 1.79 (p, *J* = 6.6 Hz, 2H). ^13^C NMR (101 MHz, DMSO-d_6_) δ 165.3, 165.3, 164.6, 152.9, 152.8, 141.9, 140.9, 131.8, 124.9, 124.6, 124.6, 106.7, 106.7, 70.7, 62.0, 60.2, 56.2, 56.1, 35.8, 29.8. LC–MS (ESI +) *m*/*z* calcd. for C_35_H_49_N_3_O_11_ [(M + H)]^+^: 688.34; found: 688.30. HPLC *t*_R_: 7.254 min.

**3-(4-(3-((4-(3-isothiocyanatopropoxy)-3,5-dimethoxybenzoyl)oxy)propyl)piperazin-1-yl)propyl 3,4,5-trimethoxybenzoate (14c).** Intermediate **13b** was dissolved in DCM (0.2 M) and allowed to cool down to 0 °C after which TFA (20 equiv) was added dropwise. The mixture was stirred for 4 h, concentrated *in vacuo* and co-evaporated with toluene to remove the excess TFA. The obtained deprotected amine (171 mg, 0.18 mmol, 1.0 equiv) was dissolved in THF (1.8 mL) and the stirred solution was allowed to cool down to 0 °C. Subsequently triethylamine (0.07 mL, 0.53 mmol, 3.0 equiv) and thiophosgene (0.02 mL, 0.19 mmol, 1.1 equiv) were added dropwise and the reaction mixture stirred for 0.5 h at 0 °C under N_2_ atmosphere. The mixture was diluted with DCM (40 mL) and subsequently washed with H_2_O (40 mL). The aqueous phase was extracted twice with DCM (40 mL) after which the organic phases were combined, dried over MgSO_4_, filtrated and concentrated *in vacuo*. Flash column chromatography on silica gel using a gradient of 3 to 5% MeOH in DCM as mobile phase provided final compound **14c**. Yellow oil (12.0 mg, 0.02 mmol, 10%). ^1^H NMR (400 MHz, CDCl_3_) δ 7.29 (s, 2H), 7.28 (s, 2H), 4.41 – 4.33 (m, 4H), 4.12 (t, *J* = 5.6 Hz, 2H), 3.91 (s, 15H), 3.91 – 3.85 (m, 2H), 2.54 – 2.40 (m, 12H), 2.14 – 2.04 (m, 2H), 2.03 – 1.92 (m, 4H). ^13^C NMR (101 MHz, CDCl_3_) δ 166.4, 166.3, 153.2, 153.1, 140.7, 125.9, 125.5, 106.9, 106.7, 69.2, 63.8, 63.7, 61.1, 56.4, 56.4, 55.2, 53.3, 41.9, 30.8, 26.4. LC–MS (ESI +) *m/z* calcd. for C_33_H_45_N_3_O_10_S [(M + H)]^+^: 676.29; found: 676.25. HPLC *t*_R_: 8.371 min.

**3-(4-(3-((3,5-dimethoxy-4-(3-(vinylsulfonamido)propoxy)benzoyl)oxy)propyl)piperazin-1-yl)propyl 3,4,5-trimethoxybenzoate (14d).** Intermediate **13b** was dissolved in DCM (0.2 M) and allowed to cool down to 0 °C after which TFA (20 equiv) was added dropwise. The mixture was stirred for 4 h, concentrated *in vacuo* and co-evaporated with toluene to remove the excess TFA. The obtained deprotected amine (240 mg, 0.38 mmol, 1.0 equiv) was dissolved in DCM (8.0 mL) and the stirred solution was allowed to cool to 0 °C. Triethylamine (0.26 mL, 1.89 mmol, 5.0 equiv) and 2-chloroethane-1-sulfonyl chloride (0.05 mL, 0.45 mmol, 1.2 equiv) were added and the reaction mixture stirred for 0.5 h at 0 °C. The reaction was quenched with 20 mL of sat. NH_4_Cl solution and subsequently extracted three times with DCM (30 mL). The combined organic phases were dried over MgSO_4_, filtrated and concentrated *in vacuo*. Automatic column chromatography (C18) with 0 to 90% CH_3_CN in H_2_O + 0.1% TFA as mobile phase on Biotage® Selekt provided final compound **14d**. Transparent oil (58.0 mg, 0.08 mmol, 21%). ^1^H NMR (400 MHz, CDCl_3_) δ 7.26 (s, 2H), 7.25 (s, 2H), 6.54 (dd, *J* = 16.6, 9.9 Hz, 1H), 6.24 (d, *J* = 16.6 Hz, 1H), 6.19 (br s, 1H), 5.92 (d, *J* = 9.9 Hz, 1H), 4.40 (t, *J* = 5.9 Hz, 4H), 4.18 (t, *J* = 5.5 Hz, 2H), 3.92 (s, 6H), 3.91 (s, 9H), 3.63 (s, 8H), 3.34 – 3.27 (m, 2H), 3.25 – 3.17 (m, 4H), 2.31 – 2.19 (m, 4H), 1.99 (p, *J* = 5.8 Hz, 2H). ^13^C NMR (101 MHz, CDCl_3_) δ 166.2, 166.0, 163.0, 162.6, 153.1, 152.8, 142.7, 141.0, 136.3, 126.2, 124.7, 124.4, 117.6, 114.7, 72.1, 61.5, 61.4, 61.1, 56.4, 56.3, 54.5, 54.5, 48.9, 48.8, 41.6, 29.1, 23.9. LC–MS (ESI +) *m*/*z* calcd. for C_34_H_49_N_3_O_12_S [(M + H)]^+^: 724.31; found: 724.25. HPLC *t*_R_: 7.548 min.

### [^3^H]NBTI displacement assays

Dilazep and ST7092 were obtained from ASTA-Werke AG (Bielefeld, Germany) and Chemie Linz AG (Linz, Austria), respectively. Radioligand displacement assays were performed as previously described [11]. In brief, the experiments were performed on erythrocyte membranes (Sanquin Bloedvoorziening, Amsterdam, The Netherlands) using a single concentration of 1 µM or multiple concentrations (ranging from 10^–10^ to 10^–6^) of unlabeled inhibitors in the presence of 4 nM [^3^H]NBTI at 10 °C for 1 h in assay buffer (50 mM Tris–HCl, pH 7.4, 0.1% w/v CHAPS). Non-specific binding (NSB) was assessed with 10 µM unlabeled NBTI. To characterize time-dependent affinity, erythrocyte membranes were preincubated with potentially covalent inhibitors for 4 h at 10 °C. Subsequently, [^3^H]NBTI was added to the preincubated and control samples (containing erythrocyte membranes, unlabeled inhibitor and radioligand) and both types of samples were incubated for another hour. Incubation was terminated by rapid vacuum filtration over Whatman™ UniFilter™ 96-well GF/C microplates using a FilterMate™ Universal Harvester (PerkinElmer, Groningen, The Netherlands). Subsequently, the plates were washed ten times with ice-cold wash buffer (50 mM Tris–HCl, pH 7.4). Thereafter, the filters were dried for 45 min at 55 °C and 25 µL of Microscint (PerkinElmer) was added per well. The filter-bound radioactivity was determined by scintillation spectrometry using a MicroBeta^2^ 2450 Microplate Counter (PerkinElmer).

### Wash-out [^3^H]NBTI displacement assays

Wash-out assays (as described previously [11]) were performed by incubation of competing inhibitors at a final concentration of 10 × K_i_ (as determined in displacement assays) with erythrocyte membranes at 10 °C for 1 h while gently shaking. Subsequently the samples were centrifuged at 13,200 rpm (16,100 *g*) at 4 °C for 5 min and the supernatant containing the unbound inhibitor was aspirated. Pellets were resuspended in 1 mL of assay buffer (50 mM Tris–HCl, pH 7.4, 0.1% w/v CHAPS), and samples were incubated at 10 °C for 10 min. After four cycles of centrifugation and washing, the supernatant was discarded and the membranes were resuspended in a total volume of 400 μL containing 4 nM [^3^H]NBTI and subsequently incubated at 10 °C for 60 min. Incubations were terminated by rapid vacuum filtration through GF/C filters using a Brandel harvester (Brandel, Gaithersburg, MD, USA). Filters were washed three times with ice-cold wash buffer (50 mM Tris–HCl, pH 7.4) and collected in tubes. Emulsifier-Safe™ scintillation fluid (PerkinElmer) was added and samples were counted by scintillation spectrometry using a Tri-Carb 2900TR liquid scintillation analyzer (PerkinElmer, Boston, MA, USA). All experimental data was analyzed using GraphPad Prism 10.0.2 software (GraphPad Software Inc., San Diego, CA, USA). [^3^H]NBTI assays were baseline-corrected with NSB and normalized to this value (0%) and washed or unwashed TB (100%), respectively. Data shown represent the mean ± SD of three individual experiments each performed in duplicate. Differences between unwashed and washed was determined in an unpaired Student’s t-test with Welch’s correction. Significant differences are displayed as * p < 0.05.

### Data analysis

For data analysis, dilazep and ST7092 were used as positive controls. All experimental data was analyzed using GraphPad Prism 10.0.2 software (GraphPad Software Inc., San Diego, CA, USA). [^3^H]NBTI assays were baseline-corrected with NSB and normalized to this value (0%) and TB (100%). The radioligand displacement curves were fitted to a one-site binding model by nonlinear regression from which the pIC_50_ values were obtained. Additionally, pK_i_ values were calculated from pIC_50_ values and the saturation K_D_ value (1.1 nM [11]) via the Cheng-Prusoff equation [19]: K_i_ = IC_50_/(1 + [radioligand]/K_D_). Data shown represent the mean ± SD of at least two or mean ± SEM of at least three individual experiments each performed in duplicate.

### Molecular docking

The binding poses of dilazep derivatives on hENT1 were predicted using molecular docking with ICM-Pro version 3.9-2c (Molsoft LLC, San Diego). Protein and ligand preparation was previously done by converting ENT1 X-ray structure co-crystalized with NBTI (PDB: 6OB6) and dilazep (PDB: 6OB7) to ICM objects. The proteins were prepared by adding and optimizing the position of hydrogen atoms, as well as the orientation and protonation states of histidine and cysteine residues and the orientation of glutamine and asparagine residues. Moreover, the stabilizing mutations F168L and A175P were reverted. Prior to docking, 6OB6 and 6OB7 were aligned based on their 3D structure to superimpose the location of NBTI and dilazep. Protonation states of the ligands to be docked (dilazep, ST7092, **6m**, **6n**, **10b**, **10e**, and **14b**) were computed, resulting in monoprotonated and unprotonated (homo)piperazine rings. All protonation states of the homopiperazine and piperazine rings were kept for docking, but only the most biologically active unprotonated state [18] was considered for further analysis. Docking was performed on ICM-Pro with default settings after generating receptor maps with both dilazep and NBTI as the center of the binding pocket in the 6OB7 structure. This resulted in a binding pocket of 48 residues. Three poses were generated for each of the ligands with a thoroughness of 3.00. Furthermore, compound **10b** was covalently docked in ICM-Pro with custom covalent reactions targeting C439. The results from docking were subsequently filtered and analyzed considering the predicted docking scores and interactions, as well as the available experimental data. The best poses in terms of docking score that maintained the general binding mode of dilazep were selected for further analysis and were visualized using PyMOL version 2.5.2 [20]. For visualization purposes, residues 440–451 in TM2 were hidden in the representations.

## Results

### Design and synthesis

**Scheme 1:**
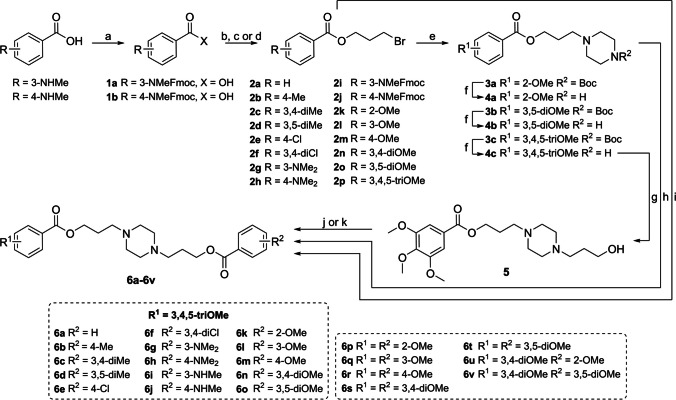
Synthesis of dilazep derivatives **6a**-**6v**. Reagents and conditions: a) Fmoc-Cl, K_2_CO_3_, dioxane, 0 °C, 21–41 h, 28–38%; b) 3-bromopropan-1-ol, Et_3_N, DCM, 0 °C to rt, 6–24 h, 68%-quant.; c) i. SOCl_2_, reflux, 3 h; ii. 3-bromopropan-1-ol, Et_3_N, DCM, 0 °C to rt, 17–29 h, 21–84%; d) EDC·HCl, DMAP, DCM, rt, 20–22 h, 28–60%; e) 1-Boc-piperazine, K_2_CO_3_, KI, anhydrous DMF, rt, 3 days, 53–89%; f) TFA, DCM, 0 °C, 4 h, quant.; g) 3-bromopropan-1-ol, K_2_CO_3_, KI, anhydrous DMF, rt to 50 °C, 16 h, 65%; h) **2a-2k** or **2n**, K_2_CO_3_, KI, anhydrous DMF, rt to 50 °C, 3 days, 14–87%; i) piperazine, K_2_CO_3_, KI, anhydrous DMF, rt to 50 °C, 3 days, 15–78%; j) corresponding R^2^-benzoyl chloride, Et_3_N, DCM, 0 °C to rt, 2 h, 14–94%. k) i. corresponding R^2^-benzoic acid, SOCl_2_, reflux, 3 h; ii. Et_3_N, DCM, 0 °C to rt, 4 h, 33–73%

**Scheme 2:**
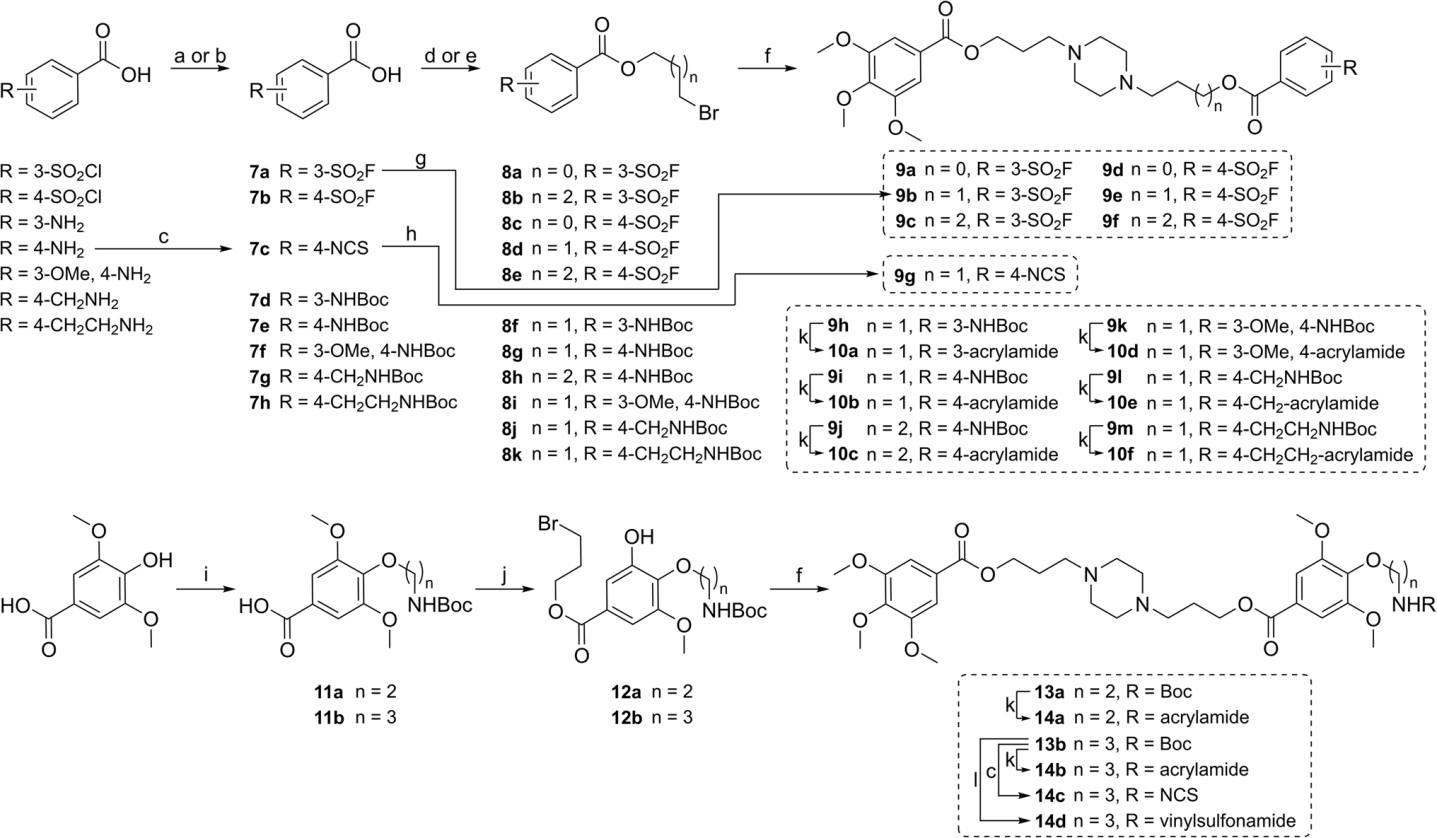
Synthesis of warhead-containing dilazep derivatives. Reagents and conditions: a) 2 M KHF_2_ (aqueous), dioxane, rt, 1 h, 94–98%; b) Boc_2_O, Et_3_N or NaOH, dioxane/water, rt, 17–25 h, 70%-quant.; c) CSCl_2_, acetone, 0 °C to rt, 16 h, 10–38%; d) i. SOCl_2_, reflux, 3 h; ii. corresponding bromoalcohol, Et_3_N, DCM, 0 °C to rt, 17–29 h, 41–100%; e) i. oxalyl chloride, cat. DMF, DCM, 0 °C, 4 h; ii. corresponding bromoalcohol, Et_3_N, anhydrous dioxane or DCM, rt, 16–23 h, 15–57%; f) **4c** (from Scheme 1), K_2_CO_3_, KI, anhydrous DMF, rt, 16 h-4 days, 2–84%; g) i. SOCl_2_, reflux, 3 h; ii. **5** (from Scheme 1), Et_3_N, DCM, 0 °C to rt, 16 h, 5%; h) **5** (from Scheme 1), EDC·HCl, DMAP, DCM, rt, 16 h, 8%; i) TBAOH, 2-(Boc-amino)ethyl bromide or 3-(Boc-amino)propyl bromide, THF, 0 °C, 2 days, 59–88%; j) EDC·HCl, DMAP, DCM, rt, 4–17 h, 39–45%; k) i. TFA, DCM, 0 °C to rt, 4 h, quant. ii. acryloyl chloride, Et_3_N, DCM, 0 °C to rt, 0.5–19 h, 8–78%; l) i. TFA, DCM, 0 °C to rt, 4 h, quant. ii. 2-chloroethanesulfonyl chloride, Et_3_N, DCM, 0 °C, 0.5 h, 21%

Several compounds were designed and synthesized with different substitution patterns on one or both phenyl rings of parent structure ST7092 (Scheme 1). Additionally, several potentially covalent inhibitors were designed and synthesized (Scheme 2). From early analysis of the hENT1 binding pocket, C439 emerged as potential target amino acid residue for covalent binding (supplementary Figure S1b). In order to establish an irreversible interaction, a variety of electrophilic warheads were installed at one site of the trimethoxy benzoate. All compounds synthesized in this study, generally followed a convergent synthesis approach similar as described by Playa et al*.* [17].

The synthesis of non-covalent compounds either started with substituted benzoyl chlorides or, where substituted benzoyl chlorides were not commercially available, the in situ conversion of substituted benzoic acids into their corresponding acyl chlorides using SOCl_2_, followed by a nucleophilic substitution by 3-bromopropan-1-ol to obtain bromopropyl benzoates **2a**-**2m** and **2p**. In the case of **2i** and **2j**, commercially available methylamino benzoic acids were first protected using Fmoc-Cl to yield intermediate **1a** and **1b** prior to the aforementioned acylation. Lastly, bromopropyl benzoates **2n** and **2o** were synthesized from their corresponding benzoic acids via a Steglich esterification. Three of these building blocks (**2k**, **2o** and **2p**) were subsequently substituted with 1-Boc-piperazine, followed by removal of the Boc group using TFA to give secondary amines **4a**-**4c**. With the use of intermediates **2a**-**2k**, alkylation of secondary amine **4c** resulted in the synthesis of final compounds **6a**-**6k**. Similarly, final compounds **6u** and **6v** were synthesized from alkylation of intermediates **4a** and **4b** using bromopropyl benzoate **2n**. Towards final compounds **6l**-**6o**, piperazine intermediate **4c** was first alkylated with 3-bromopropan-1-ol to give intermediate **5**. This was subsequently acylated with either commercially available or in situ synthesized benzoyl chlorides to give the final compounds. Finally, bis-alkylation of piperazine with the corresponding bromopropyl benzoates resulted in the synthesis of symmetrical ST7092 derivatives **6p**-**6t**.

Similar to the non-covalent inhibitors, the potentially covalent inhibitors started with the synthesis of different bromopropyl benzoate building blocks (Scheme 2). Firstly, fluorosulfonyl-warhead-substituted benzoic acids (**7a** and **7b**) were prepared from the commercially available chlorosulfonylbenzoic acids through treatment with an aqueous solution of 2 M KHF_2_. Subsequently, intermediates **7a** and **7b**, were used in similar conditions as mentioned before, using in situ acyl chloride-formation by thionyl chloride, followed by substitution with the corresponding bromoalcohol to synthesize bromoalkyl benzoates **8a**-**8e**. Alternatively, **7a** was also directly acylated onto intermediate **5** (Scheme 1) to furnish final compound **9b**. Using bromoalkyl benzoates **8a**-**8e** in a nucleophilic substitution on intermediate **4c** (Scheme 1) gave final compounds **9a** and **9c**-**9f**. Additionally, several commercially available amine-substituted benzoic acids were Boc-protected to give intermediates **7d**-**7h** in high yields, which were likewise converted to their corresponding bromoalkyl benzoates **8f**-**8k** through oxalyl chloride and a catalytic amount of DMF followed by bromoalcohol substitution. Alkylation of secondary amine **4c** (Scheme 1) with the use of intermediates **8f**-**8k**, gave potentially covalent inhibitor precursors **9h**-**9n** which were deprotected using TFA. Subsequently, an acrylamide warhead was installed on the formed free amine to obtain final compounds **10a**-**10f**. In the presence of thiophosgene, 4-aminobenzoic acid was converted to the isothiocyanate-substituted intermediate **7c**, which was used to obtain final compound **9g** from intermediate **5** (Scheme 1) by a Steglich esterification in presence of EDC and DMAP.

To include potentially covalent inhibitors with warheads installed on one of the existing methoxy substituents of ST7092, syringic acid was alkylated with the appropriate Boc-protected bromoalkylamine in a chemoselective manner using a solution of tetrabutylammonium hydroxide to give intermediates **11a** and **11b** [21]. Subsequent Steglich esterification of benzoic acids **11a** and **11b** with bromopropan-1-ol resulted in the bromopropyl benzoates **12a** and **12b**. Nucleophilic substitution of intermediates **12a** and **12b** with the use of secondary amine **4c** (Scheme 1) provided precursors **13a** and **13b**. To obtain acrylamide substituted final compounds **14a** and **14b**, the aforementioned conditions with TFA and acryloyl chloride were used. Finally, intermediate **13b** was similarly deprotected, followed by treatment with thiophosgene or 2-chloroethanesulfonyl chloride to provide final compounds **14c**-**14d**, bearing an isothiocyanate and vinylsulfonamide warhead, respectively.

### Pharmacological characterization

To determine the affinities of the non-covalent hENT1 inhibitors, a [^3^H]NBTI displacement assay was performed on erythrocyte cell membranes endogenously expressing the transporter. All compounds were initially screened at a concentration of 1 µM to provide the percentage displacement. If more than 75% displacement was observed, compounds were subsequently characterized in full concentration-effect curves to determine their affinity.

Dilazep and close analogue ST7092 both displayed high affinity (pK_i_ values of 9.39 ± 0.06 vs. 8.75 ± 0.04, respectively) as was observed previously in literature (Table 1) [18]. Exchanging one of the trimethoxy benzoate moieties of ST7092 with hydrophobic or electron-withdrawing substituents (compounds **6a**-**6f**) completely abolished inhibitory activity of hENT1. Therefore, the influence of hydrogen bond donating and accepting groups was examined with the use of compounds **6g**-**6j**. These *para-* and *meta*-substituted dimethylamine and methylamine inhibitors, showed low [^3^H]NBTI displacement (6% to 21%), indicating the necessity of having methoxy substituents on both sides of the molecule. Hence, the minimal requirements for methoxy substitutions were investigated since removal of one of the trimethoxy benzoates resulted in major loss of inhibitory activity of these compounds. Symmetrical compounds **6p**-**6r** as well as asymmetrical compounds **6k**, **6l**, **6t** and **6u** showed little to no displacement of [^3^H]NBTI. Other asymmetrical methoxy substitution patterns (**6m**, **6s** and **6v**) displayed around 30% [^3^H]NBTI displacement, whereas removal of one of the methoxy substituents in compounds **6n** and **6o** led to submicromolar affinities with pK_i_ values of 6.87 ± 0.03 and 6.84 ± 0.06, respectively.

**Table 1:**
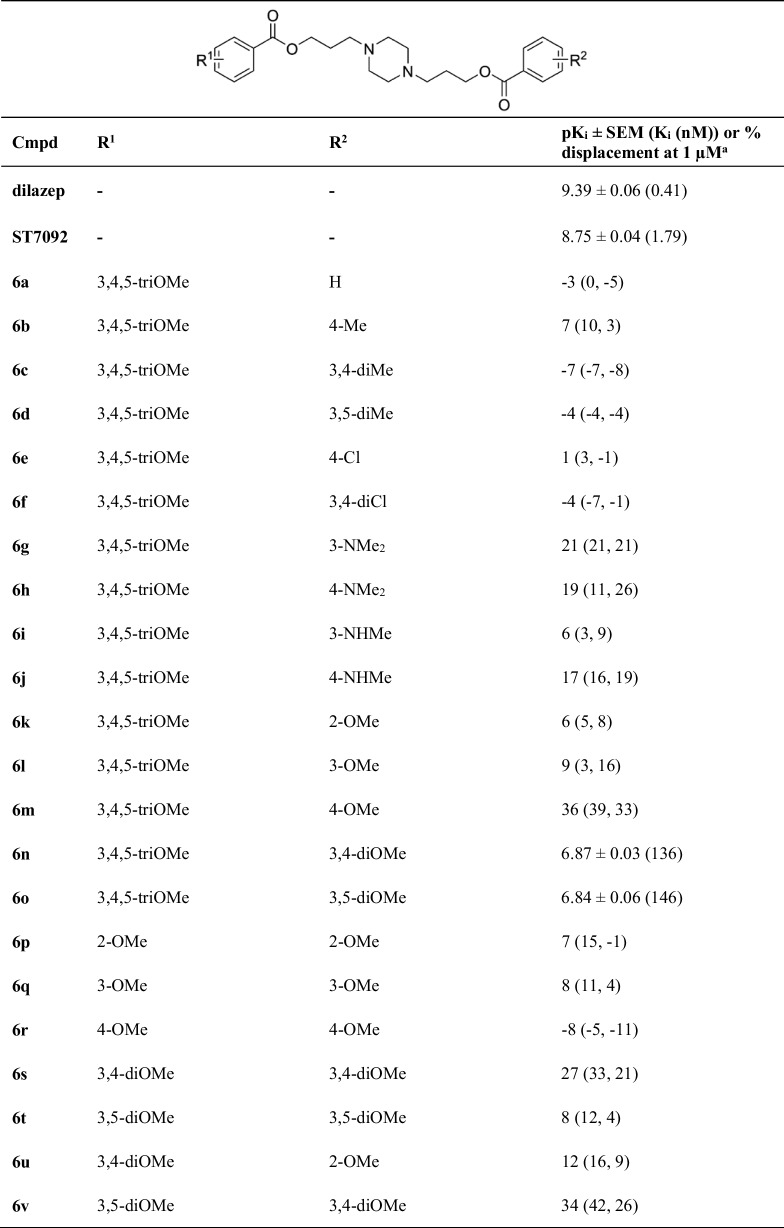
Affinity values or percentage [^3^H]NBTI displacement at 1 µM of reference inhibitors dilazep and ST7092 and compounds **6a**-**6v** in hENT1 radioligand binding assay

^a^Binding affinity (pK_i_) and percentage displacement were determined in [^3^H]NBTI displacement assays on erythrocyte membranes endogenously expressing hENT1. pK_i_ values are presented as mean ± SEM of three independent experiments performed in duplicate and percentage displacement values are mean with individual values between brackets of two independent experiments performed in duplicate.

Next, the potentially covalent inhibitors that we synthesized were subjected to the aforementioned radioligand displacement assay. To study the existence of a covalent interaction with hENT1, an additional preincubation step was included. The compounds of interest were either preincubated with the erythrocyte cell membranes for 4 h or not to determine the time-dependent shift in affinity expected upon covalent binding with C439.

Substitution of a fluorosulfonyl warhead on the para or meta position in compounds **9a**-**9f**, in combination with linker lengths between 2 and 4 carbon atoms, resulted in low [^3^H]NBTI displacement (a maximum of 61%) that seemed to slightly increase with linker length. No noteworthy differences between 4 h preincubation and without preincubation were found (Table 2). Introduction of an isothiocyanate (**9g**) resulted in decent, albeit similar affinity values with and without preincubation (7.31 ± 0.04 and 7.53 ± 0.10, respectively). Next an acrylamide warhead was introduced onto the ST7092 scaffold in various orientations (**10a**-**10f**). Binding of compound **10b** resulted in an pK_i_ value of 7.48 ± 0.15 without preincubation and a similar affinity with 4 h preincubation (7.36 ± 0.22), while close analogues **10a** and **10d** were only able to displace around 20% of [^3^H]NBTI binding. The absence of a pK_i_ shift for compound **10b** led to the design of compounds **10c**, **10e** and **10f** to accommodate a close proximity to C439. Unfortunately, increasing linker length at the alkyl chain between the piperazine- and acrylamide-substituted benzoate (**10c**) and the phenyl ring and acrylamide warhead (**10e** and **10f**) decreased hENT1 binding (between 7 and 47% displacement). In order to investigate the importance of all methoxy substituents to accommodate irreversible interaction with hENT1, an acrylamide warhead was introduced via ether linkage (compound **14a**) resulting in similar binding affinities and no observable pK_i_ shift compared to compounds **9g** and **10b**.

**Table 2:**
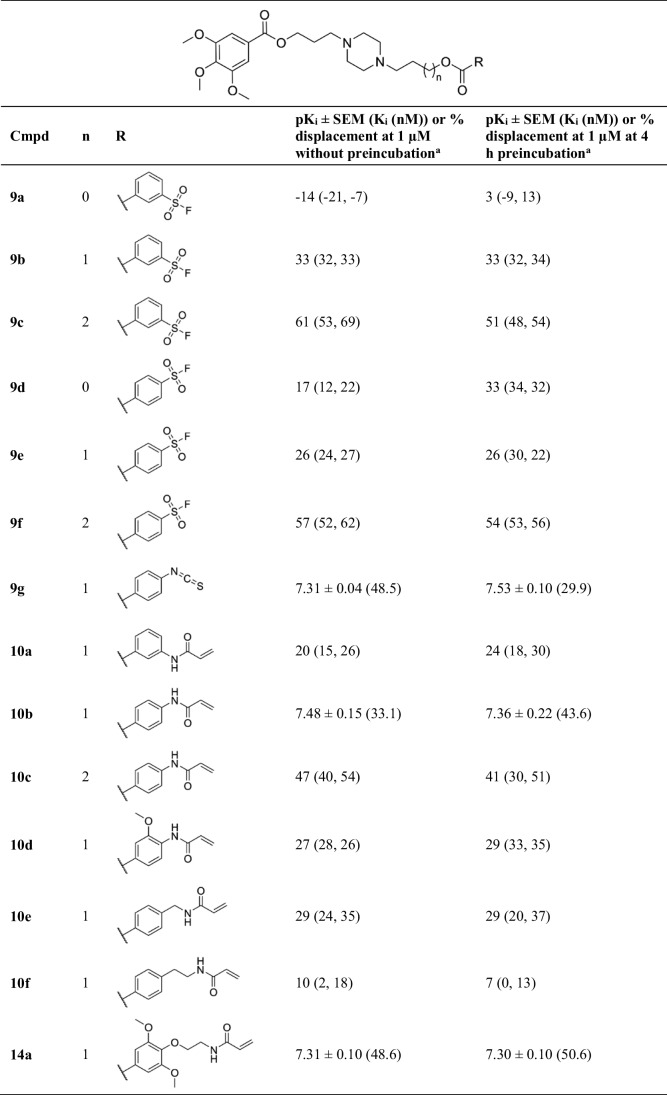

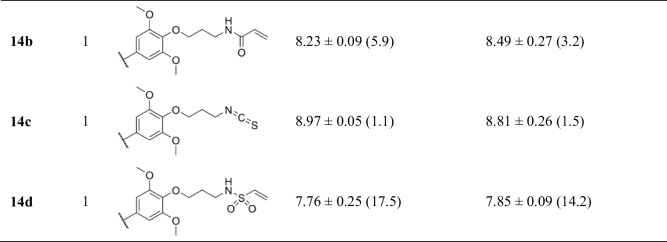
Affinity values or percentage [^3^H]NBTI displacement at 1 µM with or without 4 h preincubation of warhead-containing compounds **9a**-**9g**, **10a**-**10f** and **14a**-**14d** in time-dependent hENT1 radioligand binding assay

^a^Binding affinity (pK_i_) and percentage displacement were determined in [^3^H]NBTI displacement assays on erythrocyte membranes endogenously expressing hENT1. pK_i_ values are presented as mean ± SEM of three independent experiments performed in duplicate and percentage displacement values are mean with individual values between brackets of two independent experiments performed in duplicate

**Fig. 2:**
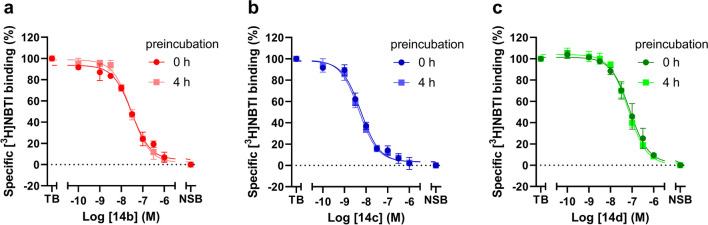
Affinity characterization of inhibitors **14b**, **14c** and **14d**. Displacement of specific [^3^H]NBTI binding by increasing concentrations of dilazep derivatives **14b** (a), **14c** (b) and **14d** (c) in erythrocyte cell membranes endogenously expressing hENT1 at 10 °C with or without 4 h preincubation. Data are normalized to 100% of the total binding and represent the mean ± SEM of three individual experiments performed in duplicate

**Fig. 3:**
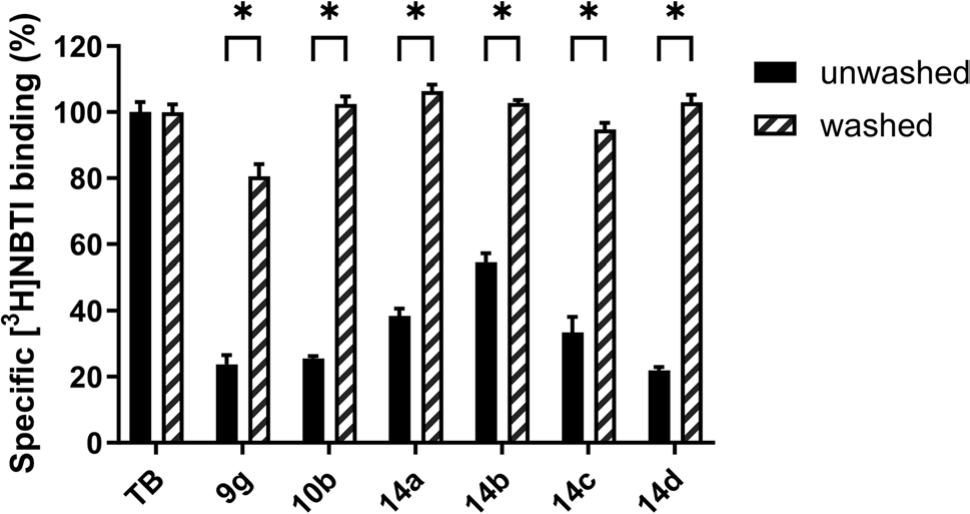
Recovery of specific [^3^H]NBTI binding after washing. Erythrocyte cell membranes endogenously expressing hENT1 were preincubated with a 10 × K_i_ concentration of ENT1 inhibitors **9g**, **10b** and **14a**-**d** for 1 h at 10 °C followed by no (unwashed) or 4 times (washed) washing. Data are shown as mean ± SD from three independent experiments performed in duplicate. Differences between unwashed and washed were determined in an unpaired Student’s t-test with Welch’s correction. Significant differences are displayed as * p < 0.05

Next, compounds **14b**-**14d**, with increased linker length and varying warheads, were tested (Fig. 2). While all three compounds showed good binding, **14b** and **14c** (Fig. 2a and 2b) containing an acrylamide and isothiocyanate warhead displayed single digit nanomolar affinities (pK_i_ values of 8.23 ± 0.09 and 8.97 ± 0.05, respectively). However, since no leftward shift in the dose response curves with 4 h preincubation was observed, no indication of irreversible binding was found for either of the three high affinity compounds. All inhibitors with an affinity below 50 nM were additionally tested in a wash-out radioligand binding assay to further investigate irreversibility of binding. All six inhibitors demonstrated a recovery of 80 to 100% of [^3^H]NBTI binding after four washing steps, indicating no covalent interaction (Fig. 3).

### Molecular docking

With the use of the published hENT1 crystal structure (PDB: 6OB7), possible binding modes of compounds ST7092, **6m** and **6n** were investigated by molecular docking.

**Fig. 4:**
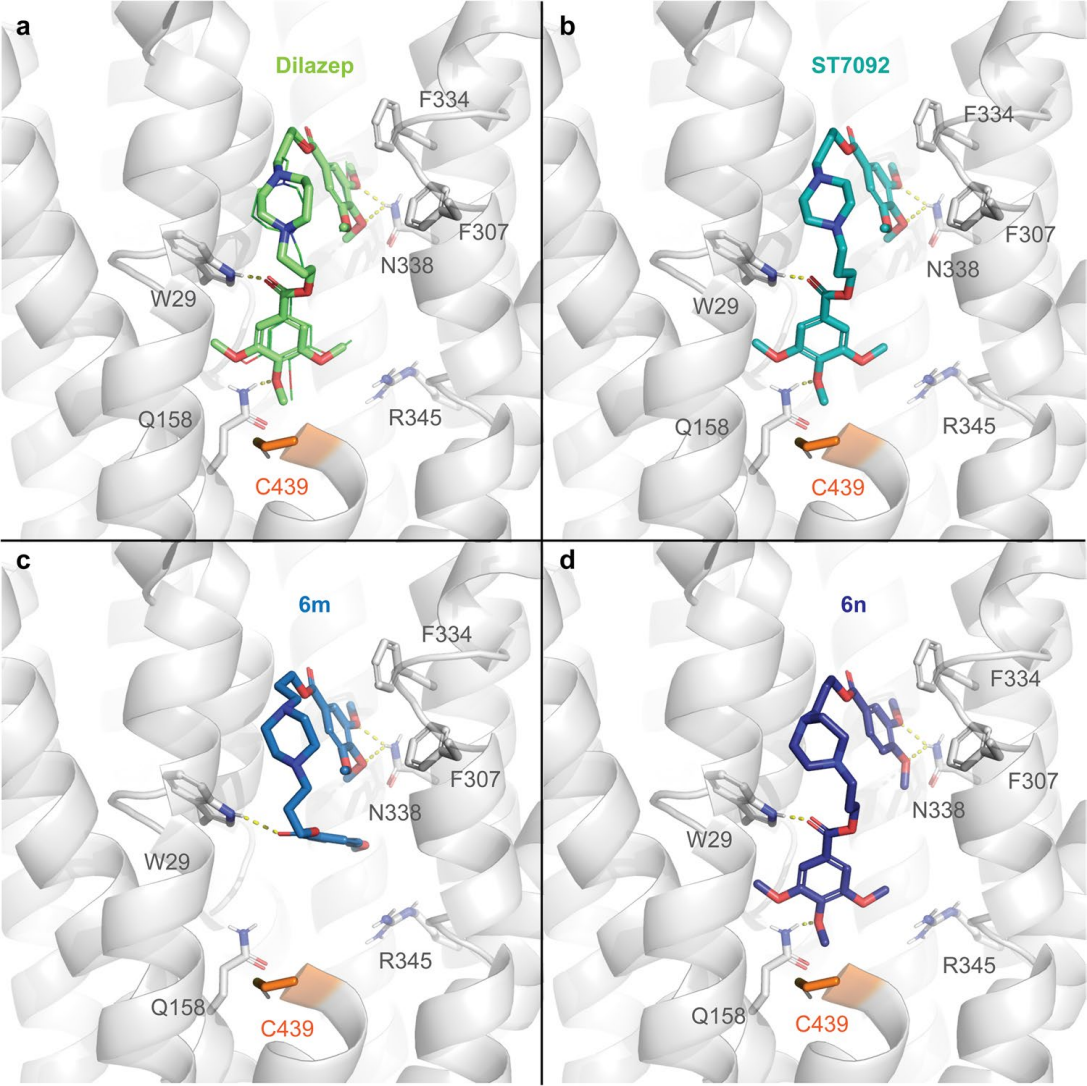
Binding poses of dilazep piperazine analogs with decreasing number of methoxy substitutions including their docking scores. hENT1 PDB: 6OB7 (grey) with (re)docked dilazep and co-crystallized structure inhibitor in a thin line for reference (a), ST7092 (b), **6m** (c), and **6n** (d). Polar contacts (hydrogen bonds) are represented as dashed yellow lines. Target nucleophilic residue C439 (TM2) is colored in orange for reference

When dilazep was redocked in the hENT1 structure, the key interactions originally described in the crystal structure were maintained, thus validating the suitability of the structure for docking of analogs (Fig. 4a). These interactions include hydrogen bonds with N338 and Q158, respectively, via the methoxy groups and a hydrogen bond with W29 via the carbonyl group that goes deeper into the pocket. As originally described, π-π stacking was observed with F334 and F307. Of note, we docked dilazep with an unprotonated homopiperazine ring, in agreement with the originally described structure. The two mono-protonated isomers also conserved the original interactions, albeit with lower docking scores. The piperazine derivative of dilazep, ST7092, maintained a similar binding pose, conserving the interactions with N338, Q158 and π-stacking at the top of the pocket (Fig. 4b). However, the change of a homopiperazine to a piperazine ring affected the torsion angles of the ring substituents leading to slightly weaker polar contacts with W29 and Q158. This was also observed in a lower binding affinity and docking score (Table 1 and Table S1). Similarly, the di-substituted piperazine analogue **6n** was able to conserve all interactions albeit with worse docking scores either with the substituted ring interacting with N338 (most energetically favorable pose, Fig. 4d), or flipped (second most favorable pose, not shown). The effect on torsion angles driven by the ring substitution meant that interactions at the bottom of the pocket were only kept when additional methoxy groups were available for interacting with Q158. When this was not the case, the interaction was lost, as it was observed for the mono-substituted analog **6m** (Fig. 4c), which was not able to interact with Q158 in any of the analyzed poses.

Six out of the seventeen compounds equipped with a warhead displayed submicromolar affinity (compounds **9g**, **10b**, **14a**-**14d**). In order to investigate their ability to displace [^3^H]NBTI, but their inability to bind covalently to C439 of hENT1, their potential binding mode was examined.

**Fig. 5:**
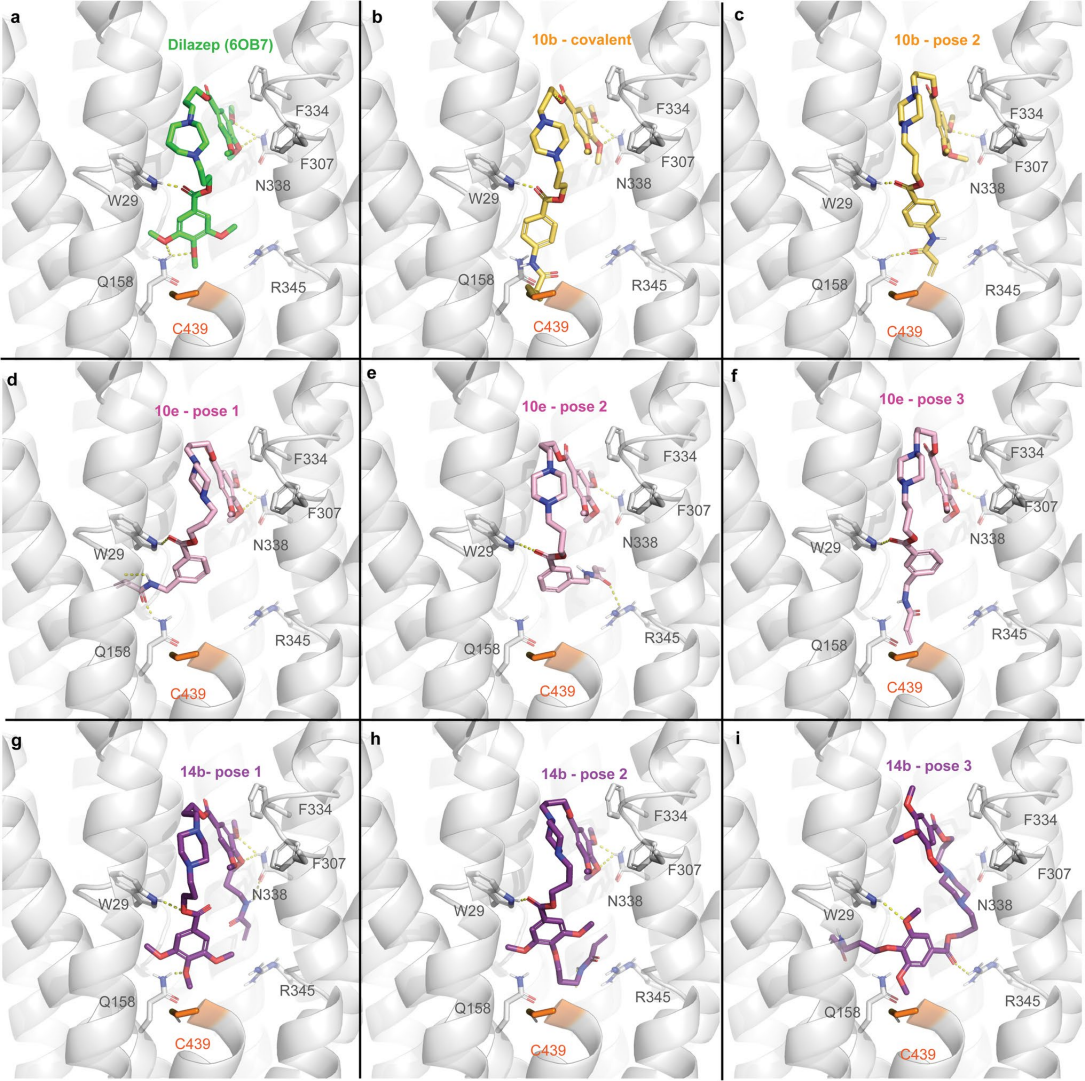
Binding poses of dilazep piperazine analogues substituted with electrophilic warheads. hENT1 (grey) crystal structure 6OB7 in complex with dilazep (a) and different docking poses of **10b** (b, covalent and c, non-covalent), **10e** (d-f) and **14b** (g-i). Polar contacts (hydrogen bonds) are represented as dashed yellow lines. Target nucleophilic residue C439 (TM2) is colored in orange for reference

Compound **10b** was used as starting point as in theory it was able to keep key interactions of dilazep with W29 and N338 while forming a covalent bond (Fig. 5b). However, given the experimental data we hypothesize that instead a hydrogen bond was formed between the warhead and Q158 (Fig. 5c). Compounds such as **10e**, with an additional carbon atom before the warhead, were not able to maintain all the key interactions while occupying the bottom part of the pocket (Figs. 5d-f). In contrast, the compounds with the warhead placed directly on the trimethoxy benzoate group, such as **14b**, showed the highest binding affinity (Table 2). From a structural point of view, this can be explained by the fact that the warhead can be accommodated at different locations in the pocket (Fig. 5g-i), including the NBTI pocket (Fig. 5i) while maintaining the canonical interactions or any other way occupying the bottom part of the pocket. Unfortunately, while the location of the warheads seemed optimal for covalent binding in several compounds (Fig. 5c, 5f, 5h), covalent interaction was not observed. Further investigation of the binding pocket to find potential influences on C439’s ability to binding covalently showed that this residue is situated in a highly hydrophobic area (supplementary Figure S1c) which consists of its adjacent neighboring residues L438 and L440 and multiple other apolar amino acids such as glycines, alanines and phenylalanines.

## Discussion and conclusion

As mentioned in the introduction, hENT1, an important therapeutic target, plays a key role in transporting adenosine, purines and nucleoside-derived drugs [22, 23]. While high affinity adenosine reuptake inhibitors like NBTI, draflazine and dipyridamole have been thoroughly investigated, studies regarding derivatives of the vasodilator dilazep are limited [24]. Moreover, molecular tools for studying the protein’s function and binding mechanisms focus mainly on NBTI- and dipyridamole-based tools [25, 26]. Hence, this study focused on the design, synthesis, pharmacological and computational evaluation of 39 novel dilazep-derived inhibitors of which17 derivatives were equipped with an electrophilic warhead aimed to establish covalent interaction with hENT1 amino acid residue C439. With the use of a [^3^H]NBTI binding assay, the binding affinities or percentage displacement of all synthesized inhibitors were determined. Pentamethoxy-substituted compounds **6n** and **6o** displayed an 80-fold decrease in affinity (Table 1) compared to ST7092, whereas further removal of methoxy groups (compounds **6k**-**6m** and **6q**-**6r**, Table 1) completely diminished [^3^H]NBTI displacement. This indicates that at least two methoxy substituents are needed at the benzoate site where alterations in the substitution pattern are made. Moreover, taken together with other investigated substitution patterns (Table 1) this demonstrates the small range of chemical diversity that is tolerated for the hENT1 as was shown previously for dilazep derivatives [17]. Although from previous research it was postulated that a single methoxy substitution on both benzoates would still be feasible [17], our research shows that symmetrical compounds with mono- or di-methoxy substitution patterns lost inhibitory activity. Furthermore, the previous study showed that substituting the trimethoxy pattern on both phenyl rings in the parent structure with a single electron withdrawing group such as fluor on the para-positions, maintained inhibitory properties at 10 µM whilst on the contrary the 4-Cl substituted **6e** was not able to inhibit [^3^H]NBTI binding at 1 µM. Additionally, the limited tolerance for chemical diversity accounts for hENT1 inhibitors in general which was found using kinetic and thermodynamic radioligand binding studies [27]. This previous research has shown that various inhibitors (dilazep, dipyridamole and NBTI) maintained a single binding mode at different temperatures which was favored by polar interactions suggesting a low tolerability for chemically diverse inhibitors which would favor different interactions. This supports our finding that most changes in the substitution pattern of ST7092 dramatically decreased affinity.

In order to gain more insight into the loss of affinity as a result of different substitution patterns on one of the ST7092 phenyl rings, compounds ST7092, **6m** and **6n** were docked into the hENT1 structure co-crystallized with dilazep (PDB: 6OB7, Fig. 4b-d) [16]. Compared to the binding mode of dilazep and ST7092, compound **6n**, where one of the *ortho*-methoxy groups of ST7092 was removed, retained similar interactions, however with weaker polar interactions with W29 and Q158 (Fig. 4d). In addition, *para*-methoxy substituted **6m** (Fig. 4c), lost interaction with Q158 in all analyzed binding poses. This compound also showed the weakest affinity, suggesting that a relatively strong interaction with Q158 is needed to displace NTBI, which uses this glutamine as its anchor point [16], followed by interactions with the other aforementioned residues (W29, F307, F334 and N338), as has been previously described for dilazep [16, 28, 29]. Accordingly, earlier mutagenesis studies have shown that mutations of these highly conserved residues significantly decreased the inhibitory properties of dilazep as well as other inhibitors NBTI, dipyridamole and draflazine [30, 31].

In multiple other protein families as well as SLC subfamilies, the use of covalent inhibitors has shown to be of great use in the field of chemical biology to determine protein expression levels and elucidate protein structures [32, 33]. Additionally, multiple covalent therapies are on the market showing advantages such as improved efficacy and decreased drug resistance [34]. Therefore, several potentially covalent inhibitors were synthesized to target cysteine 439 (TM2) in the central cavity of the binding pocket (supplementary Figure S1). To establish a first indication of covalent binding, a time-dependent radioligand binding assay was performed as previously described for other radioligand binding assays [35]. When comparing apparent affinity with 4 h preincubation to affinity values without preincubation, the apparent affinity of covalent inhibitors should increase as a result of increased transporter occupancy over time. Unfortunately, none of the potential covalent inhibitors displayed any increase in [^3^H]NBTI displacement over time at 1 µM (Table 2). However, inhibitors **14b**, **14c** and **14d** were able to displace [^3^H]NBTI at high affinity (Fig. 2). The high affinity of compound **14b** can be explained by the accommodation of the warhead in various manners (Fig. 5g-i) such as the occupation of an additional space in the binding pocket by the warhead (Fig. 5i), previously described as opportunistic site 2 [16]. This unshared binding site between inhibitors dilazep and NBTI accommodates the thioinosine moiety of NBTI through hydrophobic interactions. Unfortunately, when incorporating a 4 h preincubation step prior to the radioligand displacement assay, no significant increase of the affinity was observed for inhibitors **14b**, **14c** and **14d** (Fig. 2), indicating solely non-covalent interactions between the inhibitors and hENT1. A further series of experiments, in which we introduced washing steps to differentiate between covalently and reversibly bound inhibitors, did not yield evidence for covalent interactions either (Fig. 3).

In order to successfully design a covalent inhibitor, multiple factors should be considered (as has been extensively reviewed). Particularly important are a high affinity scaffold, a nucleophilic target amino acid residue and warhead reactivity [36]. With the rational design of the dilazep-derived covalent inhibitors multiple substitutions were made to establish a close proximity between the warhead and C439 while maintaining the interactions needed for high affinity. Elongation of the inhibitor in various ways (compounds **9c**, **9f**, **10c**, **10e**, **10f**, **14a** and **14b**) was introduced to bridge the 4.3 Å distance between the warhead substitution side and the nucleophilic side of the cysteine (supplementary Figure S1b). Although the addition of an extra linker should reach across the distance to the target anchor point, there was no indication of covalent binding (Table 2), which suggested a mismatch between the reactivity of the warhead and of C439. Covalent interactions with cysteine have been used in a plethora of protein studies because of its high intrinsic nucleophilicity (p*K*_a_ ≈ 8.5) [37]. With the use of Michael acceptors such as acrylamide and dithiocarbamate formation by isothiocyanates, reversible-covalent interactions can be formed with cysteine residues [37, 38]. Therefore, the non-covalent binding compounds **10a**-**10f**, **14a** and **14b** directed us to the design of covalent compounds bearing vinylsulfonamide and fluorosulfonyl warheads with an increased reactivity towards cysteines compared to the aforementioned [39]. However, the inability of the compounds with increased warhead reactivity to bind covalently to C439 could indicate that this amino acid residue is not intrinsically nucleophilic enough to establish covalent binding. As has been described for many other protein families, p*K*_a_ values of cysteines can widely vary depending on their spatial environment in the protein [40], which in our case is mainly hydrophobic (supplementary Figure S1c) and therefore might contribute to a reduction in reactivity [41]. Unfortunately, in silico calculations of cysteine p*K*_a_ values often show a lack of accuracy and generate false positives, making valuable predictions to be used in covalent inhibitor design challenging [42, 43]. Therefore, in further development of covalent dilazep-derived inhibitors the reactivity of other target nucleophilic amino acid residues as well as a matched warhead in terms of reactivity should be considered. Additionally, this research shows that, despite the presence of a high-affinity scaffold and a sufficiently reactive warhead, establishing covalent interactions with non-catalytic nucleophilic amino acid residues is not trivial.

In conclusion, different substitution patterns of the trimethoxy benzoates of dilazeps’ close analogue ST7092 led to decreased interactions in the binding pocket and therefore diminished hENT1 affinity. Conversely, compounds **14b**, **14c** and **14d** displayed high affinities for the transporter mainly by occupying the opportunistic site 2 without binding covalently to amino acid residue C439. Taken together, this study reported new dilazep derivatives active as hENT1 inhibitors and the first high affinity dilazep derivatives equipped with an electrophilic warhead that did not induce covalent binding in the hENT1 binding pocket. These results will aid in the rational and structure-based design of novel inhibitors as well as pharmacological tools to further study hENT1 function, binding mechanisms and (patho)physiological implications.

## Supplementary Information

Below is the link to the electronic supplementary material.

Supplementary file1 (DOCX 1219 KB)

## Data Availability

Data is contained within the article or supplementary material. Data not shown is available from the corresponding authors, upon reasonable request.

## Abbreviations

ENT1: Equilibrative nucleoside transporter 1
NBTI: *S*-(4-Nitrobenzyl)-6-thioinosine
NSB: Non-specific binding
SLC: Solute carrier
TB: Total binding
