# Supplementary material for: Exploring novel dilazep derivatives as hENT1 inhibitors and potentially covalent molecular tools
Source: Purinergic Signal. 2024 Jun 15;21(2):289–316. doi: 10.1007/s11302-024-10026-x (PMC12061832; doi:10.1007/s11302-024-10026-x)
Supplement: Supplementary file 1 — Supplementary file1 (DOCX 1219 KB) [file 11302_2024_10026_MOESM1_ESM.docx]

**Supplementary Information**

**Exploring novel dilazep derivatives as hENT1 inhibitors and potentially covalent molecular tools**

Majlen A. Dilweg^a^, Marina Gorostiola González^a^, Martijn D. de Ruiter^a^, Nadine J. Meijboom^a^, Jacobus P. D. van Veldhoven^a^, Rongfang Liu^a^, Willem Jespers^a^, Gerard J. P. van Westen^a^, Laura H. Heitman^a,b^, Adriaan P. IJzerman^a^, Daan van der Es^a^*

^a^Division of Drug Discovery and Safety, Leiden Academic Centre for Drug Research, Leiden University, PO Box 9502, Leiden, The Netherlands

^b^Oncode Institute, Leiden, The Netherlands

*Corresponding author: [d.van.der.es@lacdr.leidenuniv.nl](mailto:d.van.der.es@lacdr.leidenuniv.nl)

***Submitted to: Purinergic Signalling***

**
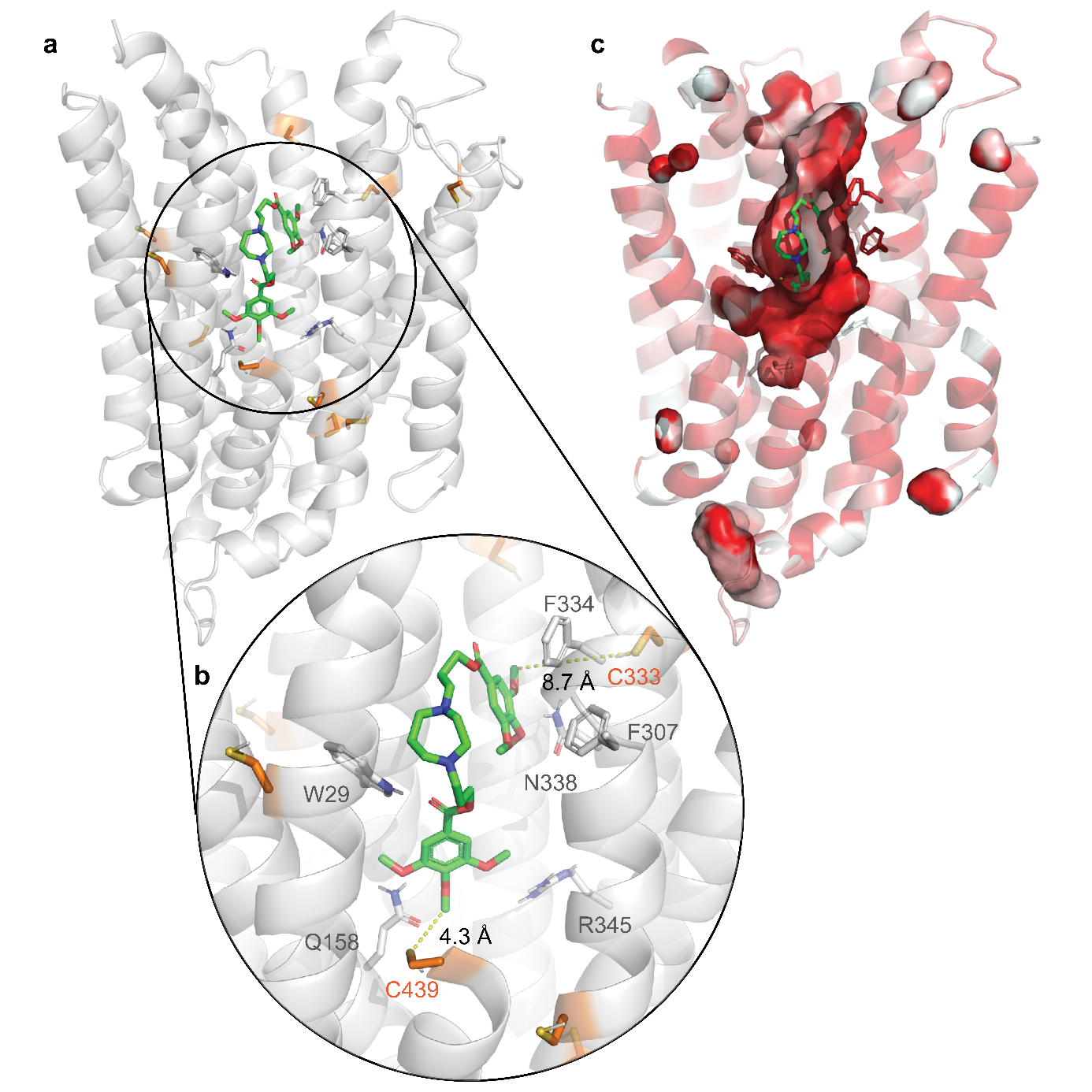
**

**Figure S1** hENT1 cysteine environment analysis for covalent docking. (a) Location of all cysteine residues in the hENT1 6OB7 prepared structure, in orange, with co-crystalized dilazep (green) for reference. (b) Distance from dilazep methoxy groups to the two closest cysteine residues, C439 and C333. (c) hENT1 binding pocket surface colored based on hydrophobicity. Darker red is more hydrophobic, white hydrophilic

**Table S1.** Docking scores and hydrogen bond network of best poses as shown in Figures 3 and 4 for dilazep derivatives in hENT1.

|  | **Docking score** | **Hydrogen bond network** | | |
| --- | --- | --- | --- | --- |
| **Compound** | **Unprotonated (homo)piperazine ring** | **W29** | **Q158** | **N338** |
| **dilazep** | -41.75 (pose 1) | + | + | ++ |
| **ST7092** | -40.14 (pose 1) | + | + | ++ |
| **6m** | -29.41 (pose 1) | + |  | ++ |
| **6n** | -35.95 (pose 1) | + | + | ++ |
| **10b** | -26.66 (covalent)  -33.98 (pose 2) | +  + | + | ++  + |
| **10e** | -27.83 (pose 1)  -27.23 (pose 2)  -23.67 (pose 3) | +  +  + | +  R345 | ++  +  + |
| **14b** | -29.41 (pose 1)  -23.70 (pose 2)  -21.37 (pose 3) | +  +  + | +  R345 | ++  ++ |
